# Multiple N-linked glycosylation sites critically modulate the synaptic abundance of neuroligin isoforms

**DOI:** 10.1016/j.jbc.2023.105361

**Published:** 2023-10-20

**Authors:** Orion Benner, Thomas P. Cast, Laurie S. Minamide, Zephyr Lenninger, James R. Bamburg, Soham Chanda

**Affiliations:** 1 Biochemistry & Molecular Biology, Colorado State University, Fort Collins, USA; 2 Molecular, Cellular & Integrated Neurosciences, Colorado State University, Fort Collins, Colorado, USA; 3 Cell & Molecular Biology, Colorado State University, Fort Collins, Colorado, USA

**Keywords:** post-translational modification, N-linked glycosylation, neuroligins, synaptic cell adhesion, protein trafficking

## Abstract

In recent years, elegant glycomic and glycoproteomic approaches have revealed an intricate glycosylation profile of mammalian brain with enormous spatial and temporal diversities. Nevertheless, at a cellular level, it is unclear how these post-translational modifications affect various proteins to influence crucial neuronal properties. Here, we have investigated the impact of N-linked glycosylation on neuroligins (NLGNs), a class of cell-adhesion molecules that play instructive roles in synapse organization. We found that endogenous NLGN proteins are differentially glycosylated across several regions of murine brain in a sex-independent but isoform-dependent manner. In both rodent primary neurons derived from brain sections and human neurons differentiated from stem cells, all NLGN variants were highly enriched with multiple N-glycan subtypes, which cumulatively ensured their efficient trafficking to the cell surface. Removal of these N-glycosylation residues only had a moderate effect on NLGNs’ stability or expression levels but particularly enhanced their retention at the endoplasmic reticulum. As a result, the glycosylation-deficient NLGNs exhibited considerable impairments in their dendritic distribution and postsynaptic accumulation, which in turn, virtually eliminated their ability to recruit presynaptic terminals and significantly reduced NLGN overexpression–induced assemblies of both glutamatergic and GABAergic synapse structures. Therefore, our results highlight an essential mechanistic contribution of N-linked glycosylations in facilitating the appropriate secretory transport of a major synaptic cell-adhesion molecule and promoting its cellular function in neurons.

The Neuroligin (NLGN) family proteins (*i.e.*, NLGN1–4) are members of postsynaptic cell-adhesion molecules (CAMs) abundantly expressed in mammalian brain (1, 2, 3). During development, NLGNs define the functional parameters of synapses in an isoform-dependent manner (4, 5, 6). Using systematic analysis of conditional and constitutive KO animals or transient overexpressions, we and others have demonstrated that NLGNs regulate the postsynaptic clustering of neurotransmitter receptors at both glutamatergic and GABAergic synapses (7, 8, 9, 10, 11, 12). In addition, loss of NLGN function by disease-associated genetic mutations can also severely disrupt the basal forms of excitatory and/or inhibitory synaptic transmission at different circuits, which further underscores their pivotal roles in neuronal communication (13, 14, 15, 16, 17, 18, 19, 20, 21, 22).

Various structural regions with unique amino acid composition are known to determine NLGNs’ mechanistic features at synapses. NLGNs form heterophilic interactions with presynaptic neurexins (NRXNs) *via* their acetyl cholinesterase (AChE)–like domains in a Ca^2+^-dependent fashion (23, 24, 25, 26, 27). Different NLGN variants also comprise multiple shared and isoform-specific sequences within their intracellular domain and extracellular domain (ECD) that enable homodimerization *versus* heterodimerization and guide their respective binding affinities for other synaptic proteins, for example, PSD-95, gephyrin, collybistin, MDGA, GARLH, and synaptic receptors (28, 29, 30, 31, 32, 33, 34, 35, 36, 37). These amino acid residues play vital roles in promoting efficient transport of NLGN isoforms to synapses, and establishing NLGN-dependent cis- and trans-synaptic molecular networks, which support an optimal assembly and organization of synapses.

NLGNs are also modulated by alternative splicing in their ECDs. Multiple splice insertions were identified for NLGN1 (A1, A2, and B sites), NLGN2 (site A), NLGN3 (A1 and A2 sites), or NLGN4 (site A), which can theoretically generate several alternatively spliced versions. These inserts directly alter NLGNs’ binding preference for NRXN, MDGA, or heparan sulphate and their synaptogenic activities at glutamatergic *versus* GABAergic synapses (38, 39, 40, 41, 42, 43, 44).

Despite a general understanding of NLGNs’ structural motifs and their functional contributions, relatively less is known about their post-translational modifications (PTMs) and how they can impact NLGNs’ synaptic profiles. NLGN1–4 have been reported to undergo diverse PTMs, including CaMKII and PKC-dependent phosphorylation, methylation, ubiquitination, both N- and O-linked glycosylation, as well as proteolytic cleavages (45, 46, 47, 48, 49, 50, 51, 52, 53, 54, 55, 56, 57). Of these, phosphorylation is perhaps the most extensively examined and best characterized PTM. Using phospho-proteomic approaches, different NLGN isoforms were found to be phosphorylated at certain serine, threonine, and tyrosine residues primarily situated in their intracellular domains (48, 49, 50, 51, 52). Eliminating these sites by mutagenesis or acute application of kinase inhibitors was shown to attenuate activity-dependent phosphorylation of NLGNs and their synaptic targeting *via* PSD-95 and gephyrin binding (48, 49, 50, 51). However, the functional importance of several other PTMs has not been broadly evaluated.

Evidently, NLGN1–4 also contain a number of asparagine residues in their ECDs with consensus sequon for potential N-linked glycosylation, which are either commonly or uniquely present in individual isoforms. A systematic peptide mapping and mass spectrometric analysis of overexpressed NLGN1 in human embryonic kidney (HEK) cells revealed the extensive carbohydrate content of its predicted glycosyl sequons (46). However, it remains unknown whether endogenous NLGNs undergo equivalent PTMs at various brain regions. Using surface plasmon resonance, it was previously reported that NLGN1 glycosylation at the N303 site (located within alternatively spliced insert B) can decrease its affinity for NRXNα/β (45). These results imply that N-glycosylation could negatively impact NLGNs’ ability to operate as a *trans*-synaptic CAM. Nevertheless, the general role of most glycosylation sites in shaping NLGNs’ functional properties remains poorly understood, especially in an *in vivo* cellular context.

In this current study, we performed a comprehensive assessment of the functional contributions of all putative N-linked glycosylation sites within the AChE domain of all major NLGN isoforms. We illustrate that (i) NLGNs are constitutively and heavily glycosylated in both mouse brain and human neurons, (ii) NLGNs obtain all three major glycan subtypes, but the endoplasmic reticulum (ER)–associated high-mannose versions are particularly essential for their reliable surface delivery, (ii) every NLGN variant receives prominent glycosylation at multiple asparagine residues, (iii) each site partially but positively modulates NLGN1–4 maturation, whereas (iv) all sites cumulatively ensure NLGNs’ proper postsynaptic trafficking *via* secretory pathway, and thus, influence their ability to recruit presynaptic terminals.

## Results

### NLGN isoforms are differentially glycosylated in mouse brain regions

NLGN1 is an important member of the NLGN family, which primarily modulates the maturation and maintenance of excitatory synapses (8, 9, 10, 29, 58). We first asked whether endogenous NLGN1 undergoes N-linked glycosylation in mouse brain *in vivo*. We dissected sections from six broadly defined brain areas of both male and female WT adult animals, homogenized them, and collected whole protein lysates. We incubated these protein extracts with PNGase F (*i.e.*, a glycosidase that removes all N-linked oligosaccharides, including both complex and hybrid, as well as immature high-mannose forms) and conducted quantitative Western blots with NLGN1-specific antibody.

**Figure 1:**
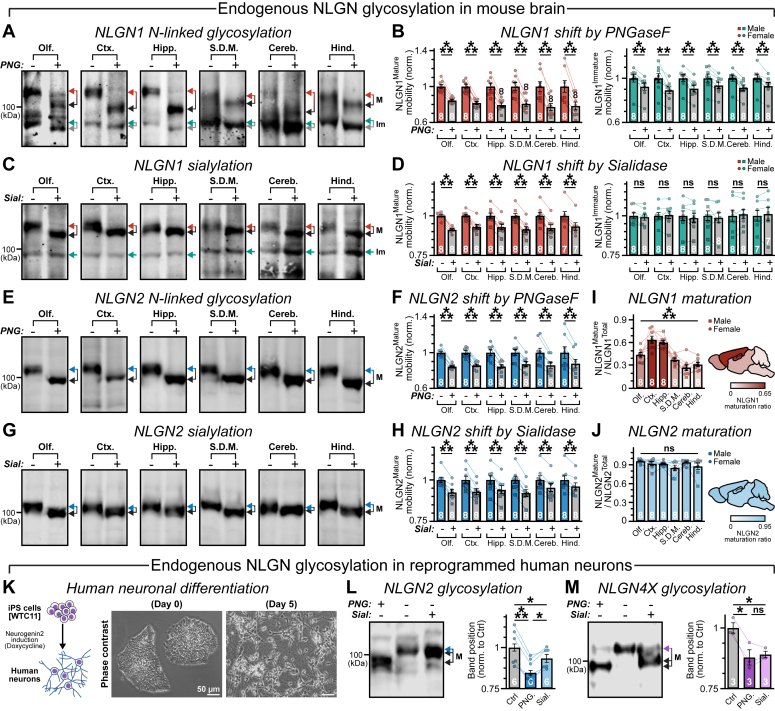
**Endogenous NLGNs are post-translationally modified by N-linked glycosylation**. *A*, representative immunoblots of endogenous NLGN1 extracted from mouse olfactory bulb (Olf.), prefrontal and cerebral cortex (Ctx.), hippocampus + dentate gyrus (Hipp.), striatum + diencephalon + midbrain (S.D.M.), cerebellum (Cereb.), and hindbrain (Hind.), before and after (“−” and “+,” respectively) incubated with PNGase F (PNG) enzyme. *Connected arrowheads* indicate PNGase F-mediated mobility shifts of NLGN1, for both maturely (labeled as “M,” *red* to *black*) *versus* immaturely (labeled as “Im,” *green* to *gray*) glycosylated products. *B*, shifts in MW by PNGase F treatment (*connected lines*) normalized to untreated controls for mature (*left panel*) *versus* immature (*right panel*) versions of endogenous NLGN1. Samples were analyzed from both male (*squares*) and female (*circles*) animals but averaged together since both genders exhibited a similar trend. *C* and *D*, same as *A* and *B*, except all samples were treated with sialidase (SIAL), which only affected the MW of maturely (*red arrowheads*) but not immaturely (*green arrowheads*) glycosylated forms of endogenous NLGN1. *E* and *H*, same as *A*–*D*, except for maturely glycosylated endogenous NLGN2 (*blue arrowheads*), when subjected to PNGase F (*E* and *F*) or sialidase (*G* and *H*) treatment. Note the limited levels of immature NLGN2 products (*E* and *G*). *I* and *J*, normalized summary plots (*bar graphs*, *left*) and *cartoon* representations (heat maps, *right*) illustrate maturely glycosylated fractions of endogenous NLGN1 (*I*) *versus* NLGN2 (*J*), at different mouse brain regions. *K*, schematic diagram (*left*) and example images of culture morphologies (*right*) of an iPS cell line (WTC-11, day 0) rapidly reprogrammed into human neurons (day 5) by doxycycline-induced Ngn2 expression. *L* and *M*, sample immunoblots (*left*) and normalized band shifts (*right*) of endogenous NLGN2 (*L*) and NLGN4X (*M*) extracted from differentiated human neurons and treated with glycosidases, that is, PNGase F or sialidase. Bar graphs represent means ± SEM, with numbers indicating total number of animals (*A*–*J*, male + female) or independent batches of human neuronal cultures (*L* and *M*) analyzed. Individual data points are plotted as *color-matched symbols*. Statistical significance was weighed using two-tailed, paired, Student’s *t* test (*A*–*H* and *L* and *M*), or one-way ANOVA (*I* and *J*), with ∗∗∗*p* < 0.005; ∗∗*p* < 0.01; ∗*p* < 0.05; ns(*p* > 0.05). iPS, isogenic-induced pluripotent stem cell line; MW, molecular weight; NLGN, neuroligin; ns, not significant.

In all untreated control samples, endogenous NLGN1 generated a ∼94 kDa product that not only nearly corresponded to its expected molecular weight (MW) but also produced a band of considerably heavier mass (size >100 kDa, Figs. 1*A* and S1*A*). We noticed that PNGase F treatments of NLGN1 caused a strong mobility shift in the higher MW products, across all brain regions for both male and female animals (Fig. 1, *A* and *B*). Therefore, these mature NLGN1 bands were substantially processed by N-linked glycosylation, regardless of gender types. These PNGase F-mediated drifts in NLGN1 size were also similarly observed for its lower MW products in every brain section analyzed, albeit by significantly lesser degrees, suggesting that they represent a relatively immature form of the protein with limited N-glycan content (Fig. 1, *A* and *B*).

We next inquired if NLGN1 is also potentially modified by sialic acid, which is often found as covalently attached to the terminal carbohydrate groups of glycoproteins. To this end, we incubated the protein extracts with sialidase (also called neuraminidase, a glycoside hydrolase that cleaves sialic acid components from both N- or O-linked glycans) and performed NLGN1 immunoblots (Fig. 1*C*). We found that sialidase application specifically reduced the size of mature NLGN1 with high MW but failed to shift its immature version with low MW (Fig. 1, *C* and *D*). Taken together, our results indicate that mouse NLGN1 is highly enriched with complex forms of glycosylation *in vivo*.

To test whether other NLGNs also receive similar PTMs, we conducted glycosidase treatments of NLGN2, a second crucial NLGN isoform that primarily controls inhibitory synaptic transmission (5, 9, 10, 31, 59, 60). Unlike NLGN1, NLGN2 predominantly existed in maturely glycosylated form (size >100 kDa), with a relatively negligible amount of its immature ∼91 kDa version (Figs. 1, *E* and *G*, and S1*A*). Once again, acute exposures to both PNGase F and sialidase effectively decreased the MW of mature bands, confirming that NLGN2 similarly obtains adequate N-glycosylation *in vivo* (Fig. 1, *E*–*H*). However, when NLGN1 and NLGN2 proteins were directly compared for the magnitude of PNGase F-mediated mobility shifts and their corresponding ratios of mature *versus* immaturely N-glycosylated bands, we observed that the cortical and hippocampal sections from both female and male animals consistently generated significantly greater level of mature NLGN1 products, whereas NLGN2 was uniformly modified across all brain areas tested (Fig. 1, *I* and *J*). Thus, depending on the isoforms, the extent of NLGN glycosylation can vary considerably between different brain regions.

### Endogenous NLGNs are constitutively glycosylated in human neurons

Of note, we could not directly assess the glycosylation pattern of NLGN3 due to the absence of a reliable antibody with minimal nonspecific signals (which otherwise obscures glycosidase-mediated MW shifts), or for NLGN4 (which is only distantly related to its human ortholog) because of its poor endogenous expression and low sequence conservation in murine brain (11, 44, 61). The X-chromosome–linked NLGN4X, however, was found to be highly expressed in human brain and neurons derived from stem cells (20, 62). To examine if NLGNs undergo N-linked glycosylation in human cellular context, we directly differentiated an isogenic-induced pluripotent stem (iPS) cell line (*i.e.*, WTC-11, containing doxycycline-inducible neurogenin-2 [Ngn2] transcription factor, (63, 64)) into neural fate (Fig. 1*K*).

Upon transgene induction, these iPS cells immediately exited from their pluripotency stage, produced neurons with elaborate dendritic arborization, and initiated synaptogenic programs as evidenced by an elevated synapsin signal (Figs. 1*K* and S2, *A*–*D*). This approach also enabled us to specifically analyze neuronal NLGNs without any source of contamination from astrocytic NLGNs in brain samples (65, 66). When probed with either NLGN2 or NLGN4X antibodies, human neurons exhibited robust endogenous expression for both proteins (Fig. 1, *L* and *M*). Moreover, acute treatments of both PNGase F and sialidase revealed substantial shift in their MWs (Fig. 1, *L* and *M*). Therefore, neuronal NLGNs are continuously processed by N-glycosylation, also in a human model.

### Cell-surface NLGNs are heavily modified by diverse glycan subtypes

**Figure 2:**
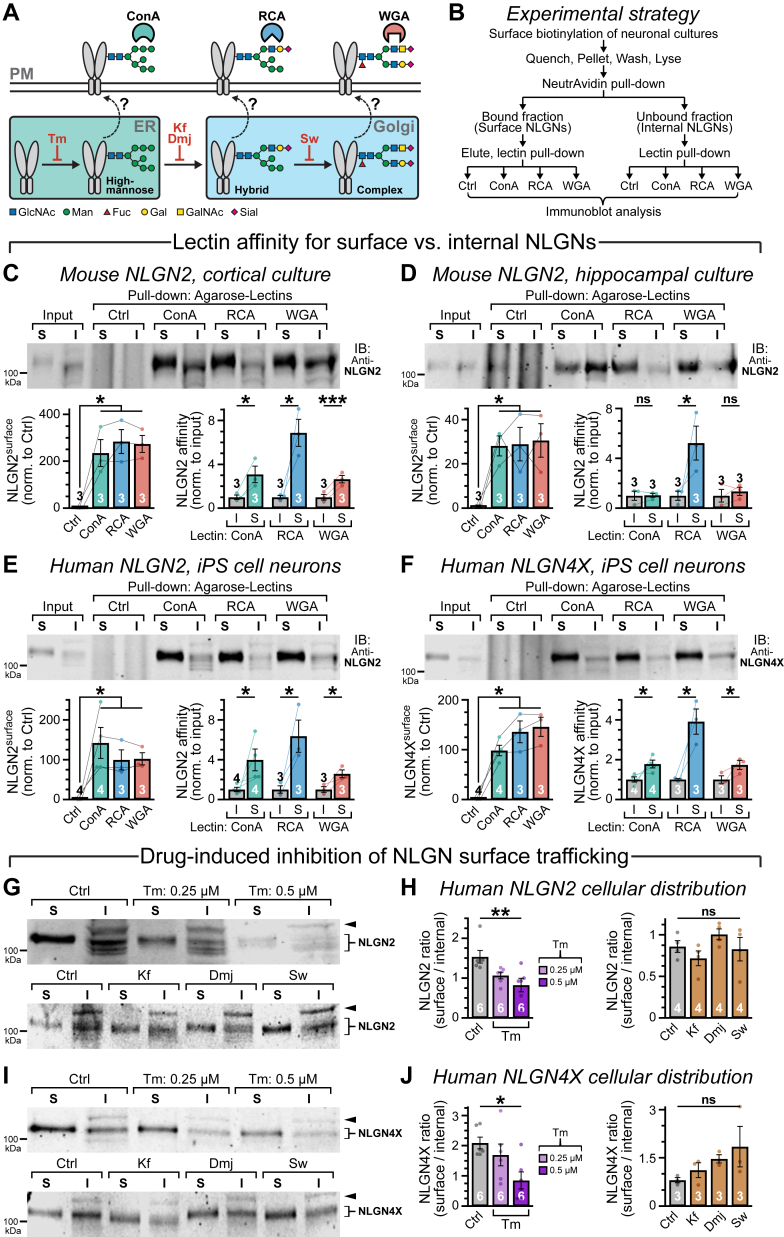
**Cell surface NLGNs display substantial heterogeneity in glycan content**. *A*, schematic diagram illustrates predicted glycosylation of NLGNs that could be recognized by specific lectins and plasma membrane (PM) transport pathways that could be potentially blocked by glycosylation inhibitors. *B*, experimental protocol to monitor the glycan subtypes of NLGNs expressed in mouse or human neurons. *C*–*F*, example of Western blots (*top*) depict surface (S) *versus* internal (I) fractions of endogenous NLGNs expressed in mouse primary neuronal cultures (NLGN2; *C* and *D*) and human iPS cell–derived neurons (NLGN2 and NLGN4X; *E* and *F*) after surface biotinylated and precipitated with lectin-conjugated agarose beads to determine their corresponding glycosylation profile. Blots were analyzed for NLGNs’ cell-surface enrichment compared with Ctrl condition (*bottom left*) or relative to their glycan content in intracellular NLGNs (*bottom right*). *G* and *H*, example of Western blots (*G*) and ratio of surface (S) *versus* internal (I) fractions (*H*) of NLGN2 extracted from iPS cell–derived human neurons that were treated with either DMSO (Ctrl) or glycosylation inhibitors, for example, Tm, Kf, Dmj, or Sw (as indicated). *Arrowheads* in *G*, a nonspecific band in internal fraction. *I*–*J*, same drug treatments as *G* and *H* but to probe surface *versus* intracellular distribution of endogenous NLGN4X. All summary plots indicate mean ± SEM, with total number (*insets* in the bar graphs) of independent batches analyzed. The values from individual samples are included as *color-matched filled circles* with *connected lines*. Statistical comparison between experimental groups was conducted using either paired, one-tailed, Student’s *t* test (*C*–*F*) or one-way ANOVA (*G*–*J*) with ∗∗∗*p* < 0.005; ∗∗*p* < 0.01; ∗*p* < 0.05; ns, *p* > 0.05. Dmj, 1-deoxymannojirimycin; DMSO, dimethyl sulfoxide; iPS, isogenic-induced pluripotent stem cell line; Kf, kifunensine; NLGN, neuroligin; ns, not significant; Sw, swainsonine; Tm, tunicamycin.

During biosynthesis, membrane proteins first receive a mannose-rich “core” glycan precursor in the ER lumen, which often matures into hybrid or complex oligosaccharides in the Golgi apparatus (Fig. 2*A*). To ascertain the glycosylation pattern of endogenous NLGNs, we biotinylated cell-surface proteins of P10–14 mouse primary neuronal cultures, avidin purified them from intracellular fraction, precipitated both by various lectins that exhibit preferential binding affinities for specific glycan subtypes, for example, concanavalin A (ConA, for high-mannose containing core N-glycans) or *Ricinus communis* agglutinin I (RCA, for *N*-acetyllactosamine [LacNAc]- and *N*-acetylgalactosamine [GalNAc]-enriched hybrid glycans) or wheat germ agglutinin (WGA, GlcNAc or sialic acid–enriched complex glycans), and immunoblotted for different NLGN isoforms to probe their relative abundance (Fig. 2*B*).

We found that NLGN2 protein localized at the cell membrane of both cortical and hippocampal cultures could be similarly recognized by ConA, RCA, and WGA, suggesting that it is likely modified by all three glycan subtypes including high-mannose, hybrid, and complex versions (Fig. 2, *C* and *D*). A comparable glycosylation pattern was also noticed for mouse NLGN1 (Fig. S1, *B* and *C*). Of the various glycan subtypes, RCA-precipitated hybrid forms were consistently associated with surface NLGN2 pool by a higher degree than its intracellular population; although both ConA-precipitated high-mannose and WGA-precipitated complex glycan types were also found to be significantly elevated in surface NLGNs, especially for the cortical cultures (Fig. 2, *C* and *D*). Moreover, membrane-bound endogenous NLGN2 or NLGN4X proteins extracted from the iPS cell-derived and astrocyte-free neurons also demonstrated evidently greater affinities for ConA, RCA, and WGA, with respect to their internal populations, confirming that surface NLGNs are enriched with diverse glycoconjugates, also in a human system (Fig. 2, *E* and *F*).

We asked whether all three glycan subtypes were essential for NLGNs’ cell-surface localization. To this end, we incubated the human neurons with tunicamycin (Tm) that blocks initial transfer of N-glycans to glycoproteins or kifunensine (Kf) and 1-deoxymannojirimycin (Dmj) that inhibit type I mannosidases necessary for hybrid and complex glycan formation, or swainsonine (Sw) that blocks type II mannosidases required for complex N-glycan formation (Fig. 2*A*). We noticed that chronic Tm application, but not Kf, or Dmj, or Sw treatments, significantly decreased the surface NLGN2 pool compared with its intracellular levels (Fig. 2, *G* and *H*). Tm applications also reduced the membrane trafficking of human NLGN4X in a dose-dependent manner (Fig. 2, *I* and *J*). This phenotype was further accompanied by reduction in NLGNs’ MWs, especially at high Tm concentrations (Fig. 2, *G* and *I*). These data suggest that the ER-associated N-linked high-mannose glycans are already sufficient for NLGNs’ maturation and could be particularly important for their appropriate secretory transport and availabilities at the neuronal membrane.

### NLGN isoforms are N-glycosylated at both unique and conserved residues

**Figure 3:**
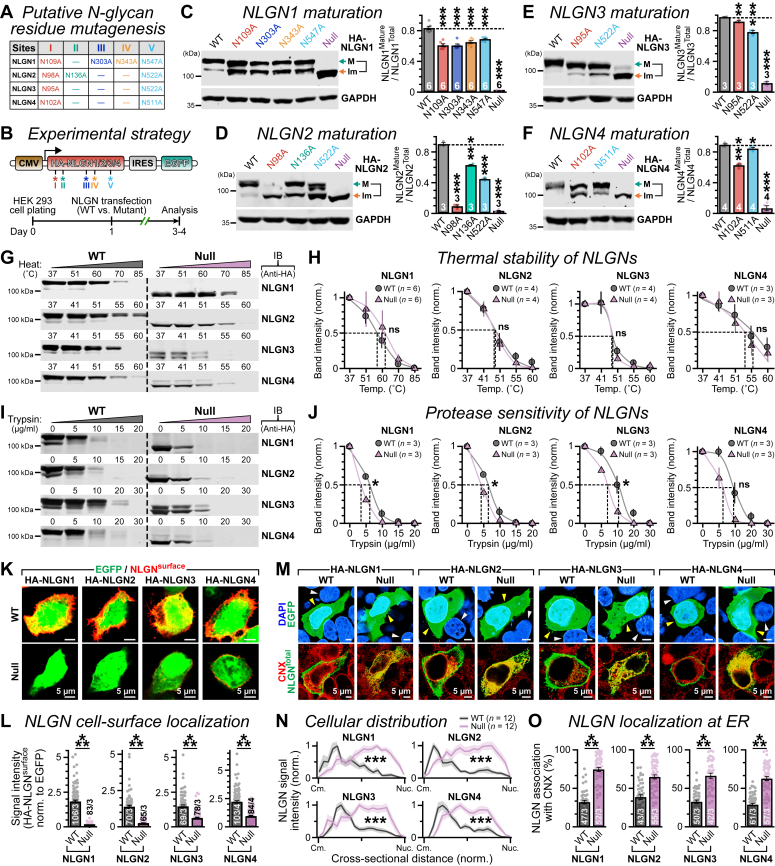
**Multipleglycosylationsites contribute toNLGNs’maturation and surface transport.***A*, the table enlists all asparagine residues at equivalent positions (unique or conserved sites I–V) that constitute consensus sequences for N-linked glycosylation within the AChE-like domains of NLGN1–4. These residues were alanine substituted, either individually (*i.e.*, single site mutants) or all together (*i.e.*, glycan-null versions) to generate the glycosylation-deficient NLGN isoforms (for details, see the Experimental procedures section). *B*, a *cartoon* representation of expression vector design (*top*) and schematic diagram of experimental timeline (*bottom*) for analyzing HA-tagged NLGN WT *versus* glycan mutant versions (*asterisks*, not to scale) in HEK cells. *C*–*F*, example of immunoblots (*left*) of maturely (labeled as “M”, *green arrows*) *versus* immaturely (labeled as “Im”, *orange arrows*) glycosylated products and average fractions of mature NLGNs (*right*, normalized to total = mature + immature), for WT *versus* glycan site mutations: NLGN1 (*C*), NLGN2 (*D*), NLGN3 (*E*), and NLGN4 (*F*). *G* and *H*, representative Western blots (*G*) and normalized band intensities (*H*) of HA-tagged NLGN1–4 WT *versus* their corresponding glycan-null versions (as labeled), when subjected to increased heat exposure. Data in *H* are fitted (*color-coded solid lines*) with Boltzmann sigmoid function (see the Experimental procedures section), and half-decay temperatures (*i.e.*, V50 values) for residual protein levels are indicated (*vertical dotted lines in black*). *I* and *J*, same as *G* and *H*, except NLGNs were digested with trypsin, and half-decay concentrations were indicated. *K* and *L*, confocal images (∼0.5 μm optical sections, *K*) of HEK cells immunostained for HA-tagged NLGN1–4 (*left* to *right*) WT *versus* corresponding glycan-null mutants, under a nonpermeabilized condition (without Triton X-100), and surface NLGN signals per cell normalized by their respective intracellular EGFP intensities (*L*). *M*, example images of HEK cells immunolabeled for total protein levels (*i.e.*, surface + intracellular) of HA-tagged NLGN1–4 WT *versus* their glycan-null variants under a permeabilized condition (with Triton X-100), coexpressed EGFP signals, and costainings for ER marker calnexin (CNX) and nuclear DAPI, as indicated. *Arrowheads* in the *top panels* point at transfected (*yellow*) *versus* nontransfected (*white*) neighboring cells. *N*, subcellular localization of NLGN1–4 WT (*gray*) *versus* glycan-deleted (*purple*) versions, as distributed between cell membrane (Cm.) and nucleus periphery (Nuc.) in transfected HEK cells (see *M*); the relative distance was sorted into 20 bins per cell radius, and signals were normalized to their maximum intensity positions. *O*, Mander’s coefficients for NLGN1–4 (*left* to *right*) colocalizing with ER marker CNX, in HEK cells (see *M*). All quantifications reflect means ± SEM, and numbers of independent experimental batches (for Western blots) or cells imaged (for immunostaining)/individual trials are reported as *insets*. All data points are included as *color-matched circles*. Statistical significances between conditions were evaluated using two-tailed, unpaired, Student’s *t* test (*C*–*J* and *O*), or two-sample Mann–Whitney *U* test (*L*), or two-way ANOVA test (*N*), with ∗∗∗*p* < 0.005; ∗∗*p* < 0.01; ∗*p* < 0.05; ns (*p* > 0.05). AChE, acetyl cholinesterase; DAPI, 4′,6-diamidino-2-phenylindole; EGFP, enhanced GFP; ER, endoplasmic reticulum; HA, hemagglutinin; HEK, human embryonic kidney cell line; NLGN, neuroligin; ns, not significant.

To evaluate how N-glycosylation influences NLGNs’ molecular properties, we aimed to map their respective PTM sites. A search for consensus asparagine sequons within AChE domains confirmed the presence of five putative glycosylation sites (termed as I–V; Figs. 3*A* and S3, *A*–*D*) located distant from their dimerization domains and transmembrane regions, which are either distinct or uniformly shared between NLGN isoforms. These included not only four sequons in NLGN1 that were previously described (45, 46) but also identified three potential sites within NLGN2, as well as two analogous sites for NLGN3 and NLGN4 proteins, which remained uncharacterized (Fig. 3*A*). We systematically substituted each of these asparagine residues to alanine either individually or when all sites combined (referred to as “null” henceforth) by a series of site-directed mutagenesis in NLGN1–4 variants and inserted hemagglutinin (HA)-epitope tags after their corresponding signal peptides (see the Experimental procedures section for details). We overexpressed these constructs in HEK 293 cells together with a cotranscribed enhanced GFP (EGFP) reporter and monitored the protein mass using immunoblots (Fig. 3*B*).

In HEK cells, WT NLGN1–4 also generated predominantly mature bands with high MW and a relatively minor level of immature bands with lower MW (Fig. 3, *C*–*F*), consistent with previous studies (20, 27). Furthermore, when exposed to PNGase F or sialidase, these mature products displayed prominent mobility shifts, which were further exaggerated by O-glycosidase (catalyzes the removal of major O-linked glycans) treatment, implying that NLGNs can undergo complex forms of glycosylation also in HEK cells (Fig. S3, *E*–*I*). We observed that all mutants lacking single N-glycosylation sites variably but significantly decreased the relative abundance of mature NLGN products when normalized to the total protein content (*i.e.*, mature + immature) and considerably elevated their immature fractions (Fig. 3, *C*–*F*). In addition, their glycosylation-deficient versions mainly produced immature bands that failed to shift when subjected to glycosidase treatments (Figs. 3, *C*–*F* and S3, *E*–*I*) and virtually eliminated NLGNs’ affinities for all lectins, ConA, RCA, or WGA (Fig. S1, *D*–*G*). These results indicate that multiple glycosylation sites collectively contribute to the final maturation of NLGN isoforms.

### N-linked glycosylation facilitates efficient cell-surface delivery of NLGNs

We sought to determine the functional relevance of N-linked glycosylation sites in NLGN1–4. N-glycosylation has previously been associated with protein folding and trafficking (67, 68, 69, 70, 71, 72). To first investigate whether deletion of N-linked glycosylation impacts NLGNs’ structural stability, we transfected HEK cells with HA-tagged WT *versus* glycan-null mutants, collected whole-cell lysates after 2 to 3 days, heated the protein extracts at variable temperatures for thermal denaturation, and quantified their aggregation-resistant portions by Western blot (Fig. 3*G*). However, we did not detect any difference between these two conditions, suggesting that glycosylation may not strongly affect NLGNs’ structural integrity (Fig. 3, *G* and *H*). To further examine any conformational differences between WT *versus* null, we digested them with trypsin of variable concentrations and measured residual fractions by immunoblot after quenching the reaction (Fig. 3*I*). Compared with WT, the null versions displayed significant but only minor increase in protease susceptibility (Fig. 3, *I* and *J*). Thus, lack of glycosylation did not critically alter NLGNs’ folding parameters. These findings are in concert with earlier work with deglycosylated NLGNs, which retained or even elevated their affinity for NRXNs *in vitro*, suggesting no major adverse effect on protein structure (40).

Since inhibition of NLGN glycosylation by Tm treatment reduced their surface levels in neurons (Fig. 2, *G*–*J*), we next inquired if removal of the N-glycosylation residues could impose similar effects. To evaluate that, we immunostained the transfected HEK cells with HA antibody under nonpermeable conditions (Fig. 3*K*). We noticed that, regardless of NLGN isoforms, all glycosylation-deficient versions showed severe impairments in their ability to localize at cell membrane (Fig. 3, *K* and *L*). This phenotype was not correlated with any change in total protein (*i.e.*, surface + internal) contents, when tested using Western blots and normalized to EGFP reporter (Fig. S4, *A*–*D*). Despite that, when immunostained under permeabilized conditions to probe for total protein, the glycan-deleted NLGN variants demonstrated preferential distribution at intracellular region away from plasma membrane edges, indicating explicit defects in their cell-surface delivery (Fig. 3, *M* and *N*).

We questioned whether NLGN1–4 mutants accumulate in secretory pathway organelles. To assess that, we colabeled the HEK cells with antibodies against both HA-NLGNs and an ER-specific marker, calnexin (Fig. 3*M*). Compared with WT NLGN1–4, the glycan-null variants triggered a pronounced rise in colocalization with calnexin (Fig. 3, *M* and *O*). Apparently, several NLGN mutants also exhibited similar enhancements in their colocalization with GM-130, a Golgi apparatus marker (Fig. S4, *E*–*H*). In sum, these data confirm that a vast majority of mutant NLGNs remain confined within internal secretory compartments leading to trafficking deficiencies.

### Glycosylation promotes NLGNs’ availability for trans-synaptic adhesion

**Figure 4:**
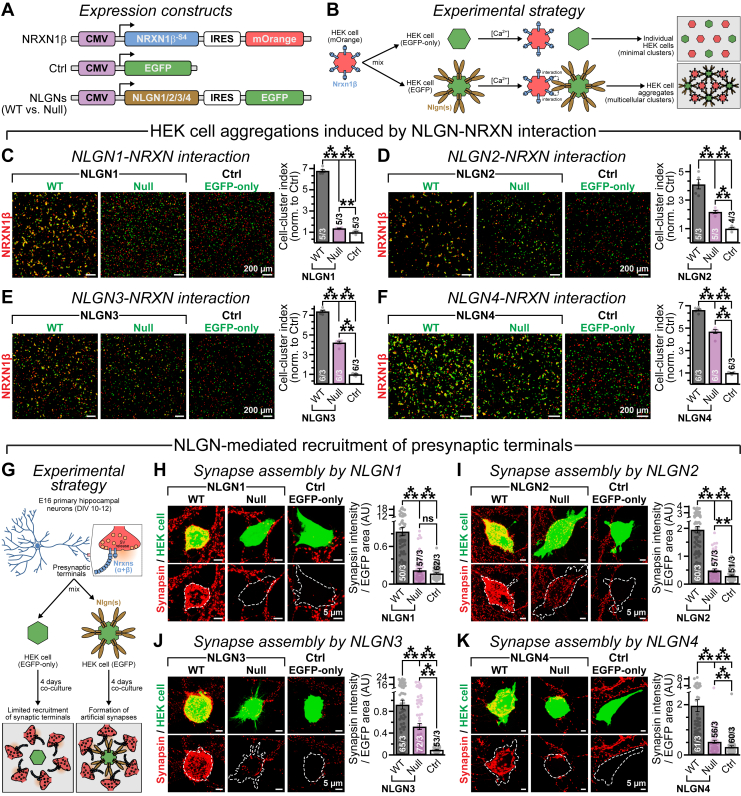
**Lack of glycosylation impairs NLGNs’ function as a trans-synaptic CAM.***A* and *B*, design of expression constructs (*A*) and schematic diagrams of experimental strategy (*B*) to probe for Ca^2+^-dependent molecular interactions between NRXN and NLGNs (WT *versus* glycan-null) at the cell surface using the HEK cell–aggregation assay (for details, see the Experimental procedures section). *C*–*F*, example images (*left*) and Mander’s colocalization coefficients (average bar graphs, *right*) of HEK cells expressing NRXN1β (mOrange labeled) mixed with cells expressing either WT *versus* glycosylation-null versions of NLGNs [NLGN1 (*C*), NLGN2 (*D*), NLGN3 (*E*), and NLGN4 (*F*)] or a control vector (Ctrl, EGFP labeled). *G*, HEK 293T cells were transfected with WT *versus* glycosylation-deficient NLGNs, or a control EGFP-only vector, and subsequently cocultured with primary hippocampal neurons derived from day 16 embryos (E16); NLGNs’ ability to recruit presynaptic terminals was monitored after 4 days (see the Experimental procedures section for details). *H*, representative images (*left*) of EGFP-positive HEK cells coexpressing NLGN1 variants (*top panels*) and surrounded by synapsin-immunolabeled presynaptic specifications from adjacent primary neurons (coinciding within *dotted outlines*, *bottom panels*); synapsin signal intensities (*right*) from different conditions, as indicated. *I*–*K*, same as *H*, except for WT *versus* glycosylation-deficient mutants of NLGN2 (*I*), NLGN3 (*J*), and NLGN4 (*K*). Summary plots are provided as means ± SEM, with total number of samples analyzed/independent batches. Individual data points are provided as *color-coded opaque circles*. Statistical assessments across conditions were performed using two-tailed, unpaired, Student’s *t* test (*C*–*F*) or two-sided, nonparametric, Mann–Whitney *U* test (*H*–*K*), with ∗∗∗*p* < 0.005; ∗∗*p* < 0.01; ns (*p* > 0.05). CAM, cell-adhesion molecule; EGFP, enhanced GFP; HEK, human embryonic kidney cell line; NLGN, neuroligin; NRXN, neurexin; ns, not significant.

Removal of N-glycans by PNGase F treatment or deletion of N-glycosylation sites by mutagenesis can markedly increase NLGN1’s affinity for NRXNs *in vitro* (45), but that does not clarify whether it can also potentiate NLGNs’ adhesion properties, especially in a cellular context. To address this issue, we transfected HEK cell cultures with NRXN1β^-SS4^ construct followed by mOrange reporter and a second population with either EGFP-only control or NLGN1–4 WT *versus* null constructs together with EGFP reporter (Fig. 4*A*). After 2 days, we mixed the NRXN- and NLGN-expressing cells and evaluated their tendency to form aggregates *via* molecular interaction (Fig. 4*B*).

We found that, with respect to EGFP controls, both WT and glycan-deficient NLGNs substantially enhanced the formation of multicellular clusters, as recognized by colocalization of mOrange and EGFP-positive cells (Fig. 4, *C*–*F*). This result suggests that glycan-null NLGNs can bind to NRXNs. Nevertheless, compared with WT NLGNs, all null conditions caused a significant reduction in cell aggregation, likely because of their diminished surface export (Fig. 4, *C*–*F*). Hence, in a cellular environment, lack of N-linked glycosylation can considerably undermine NLGNs’ capacity to engage as *trans*-synaptic CAMs, regardless of their altered affinities for presynaptic ligands.

To further validate the overall effects of glycosylation on NLGNs’ ability to ultimately interact with presynaptic terminals, we expressed WT *versus* glycan-null NLGNs in HEK cells along with an EGFP reporter, cocultured them with WT day 10 to 12 hippocampal neurons, and monitored the formation of heterologous synapses between them after 4 days by immunostaining against EGFP and presynaptic marker synapsin (Fig. 4*G*). Once again, although most null mutants were able to attract synapses on HEK cell surface more efficiently than control conditions, the magnitudes were much compromised with respect to their WT counterparts (Fig. 4, *H*–*K*). Therefore, glycosylation-dependent cell-surface trafficking of NLGNs also facilitates their apposition with presynaptic terminals *in vivo*.

### Individual glycosylation sites differentially affect NLGNs’ CAM properties

Of all glycosylation sites identified in NLGN1, the N303 residue is particularly important for modulating its NRXN affinity (45). This made us wonder if any of the unique and/or conserved glycosylation sites can also preferentially regulate the surface trafficking and synaptogenic phenotypes of different NLGN isoforms (sites I–V, Fig. 3*A*). We immunostained the HA-tagged NLGN WT *versus* single-site glycan mutants expressed in HEK cells, under nonpermeabilized conditions. We saw that, with a few exceptions (*e.g.*, site II in NLGN2 and site V in NLGN3/4), loss of glycosylation at most residues triggered a measurable decline in NLGNs’ surface export (Fig. S5, *A*–*D*). This deficit was highly consistent for all mutations at site I, which is well conserved in NLGN1–4.

Preventing N-glycosylation at individual sites also generated variable effects on NLGN-mediated recruitment of artificial synapses (Fig. S5, *E*–*H*). Notably, site I mutations again caused the most persistent decrease in synapse induction, suggesting that they impose a hierarchical impact on NLGN properties. Despite this, all single-site mutants produced relatively modest defects with respect to the glycan-null versions (Figs. 3 and 4). Thus, multiple glycosylation sites cumulatively control NLGNs’ overall maturation, trafficking, and function.

### N-glycosylation is necessary for NLGNs’ dendritic transport in neurons

**Figure 5:**
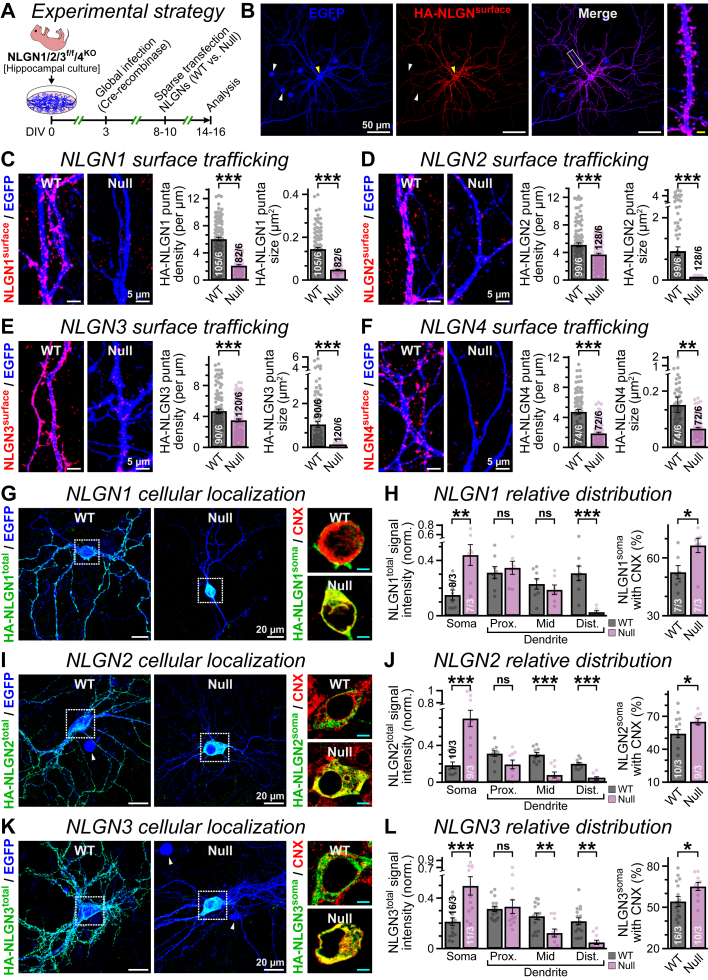
**Inhibition of N-glycosylation disrupts efficient dendritic distribution of NLGNs**. *A*, primary hippocampal neurons derived from NLGN1/2/3^flox/flox^4^KO^ mice were globally infected with lentivirus particles encoding Cre-recombinase fused to EGFP and NLS, sparsely transfected with HA-tagged NLGN1–4 WT *versus* glycan-null versions, coexpressing soluble EGFP (for details, see the Experimental procedures section). *B*, nuclei of all neurons expressing Cre-EGFP (*all arrowheads*), whereas a single cell additionally transfected with a WT NLGN variant (plus IRES-EGFP reporter) also immunostains positive for surface HA epitope (*yellow arrowhead*), under nonpermeabilized conditions. The *boxed region* from merged view is magnified to the *right*, with scale bar (*yellow*) representing 2.5 μm. *C*–*F*, example images depict EGFP-positive dendritic segments (*left*) that were immunostained for surface HA epitopes of overexpressed NLGN WT *versus* glycan-null versions, under a nonpermeabilized condition; summary plots of puncta density and size (*right*) for NLGN1 (*C*), NLGN2 (*D*), NLGN3 (*E*), or NLGN4X (*F*). *G* and *H*, maximum intensity z-projections of EGFP-positive neurons immunostained for total (surface + internal) WT *versus* glycan-null HA-NLGN1 (*G*, *left*), under permeabilized conditions, and *boxed regions* around soma (0.5 μm optical thickness) enlarged to the right with CNX counterstaining (*G*, *right*), with scale bars (*cyan*) representing 5 μm. Summary plots (*H*, *left*) depict fraction of NLGN1 confined within soma or distributed along the proximal (Prox.), medial (Mid), or distal (Dist.) regions of dendrites, and percentages of NLGN1 at soma (*H*, *right*) that are colocalized with CNX signal. *I*–*L*, same analyses as *G* and *H*, except for NLGN2 (*I* and *J*) or NLGN3 (*K* and *L*). *White arrowheads* = neighboring cells with nuclear EGFP (expressing Cre-recombinase) but without diffused EGFP (untransfected by HA-NLGN) signals. All average data are presented as means ± SEM, along with the total number of field of views (*C*–*F*) or neurons (*G*–*L*) analyzed/independent experimental batches (*insets* on bar graphs). Individual data points for average values are provided as *color-coded filled circles*. Statistical comparisons between NLGN WT *versus* glycosylation-deficient mutants were performed by either two-tailed (*C*–*F* and *H*–*L* [*left*]) or one-tailed (*H*–*L* [right]) unpaired Student’s *t* test, ∗∗∗*p* < 0.005; ∗∗*p* < 0.01; ∗*p* < 0.05; ns (*p* > 0.05). CNX, calnexin; EGFP, enhanced GFP; HA, hemagglutinin; NLGN, neuroligin; NLS, nuclear localization signal; ns, not significant.

After using HEK cells as a reduced system to establish the mechanistic implications of N-linked glycosylation in NLGNs, we next inquired how this PTM may influence NLGNs’ subcellular distribution in neurons. We prepared primary hippocampal neurons with NLGN1–4 KO genotypes where all endogenous NLGNs were acutely deleted by a nuclear Cre-recombinase (10, 11, 12, 73, 74), and transfected them with HA-tagged NLGN1–4 WT *versus* glycan-null mutants (Fig. 5*A*). This approach allowed us to avoid any homodimerizations or heterodimerizations between native and exogenous NLGNs, which could mask potential trafficking phenotypes. After 6 days, the successfully transfected neurons were readily identified by a coexpressed soluble EGFP reporter and subsequently immunostained for HA epitopes to detect overexpressed NLGN localization.

We first immunolabeled the neurons for surface HA-NLGNs under nonpermeabilized conditions (Fig. 5*B*). We observed that WT NLGN1–4 assumed an elaborate punctate pattern along the EGFP-positive dendrites (Fig. 5, *C*–*F*). Moreover, removal of all N-linked glycosylation residues resulted in a profound decline in their cluster density and size, irrespective of the NLGN subtypes (Fig. 5, *C*–*F*). These findings validate that NLGN glycosylation is an essential PTM in neurons, which critically modulates their ability to associate with dendritic membranes.

The major loss of glycosylation-deficient NLGNs from the dendritic branches made us hypothesize that they could be inappropriately localized at other cellular compartments. To examine that, we immunostained total HA epitopes under permeabilized conditions, that labeled both surface and intracellular NLGNs. We noticed that WT NLGNs were adequately translocated at both medial and distal regions of dendrites, whereas the majority of glycan-null mutants remained confined within cell bodies, with minor transport to the proximal dendrites (Fig. 5, *G*–*L*). We questioned if null mutants are incorrectly retained in the secretory compartments, as observed in HEK cells (Fig. 3, *M*–*O*). Coimmunostaining of these neurons by HA and calnexin antibodies revealed a substantially higher colocalization between ER and glycan-null mutants localized at the soma, compared with WT NLGNs (Fig. 5, *G*–*L*). Hence, glycan-null NLGNs remain largely restricted in the secretory pathway and poorly distribute in dendrites.

### Glycosylation is required for NLGNs’ synaptic recruitment and function

**Figure 6:**
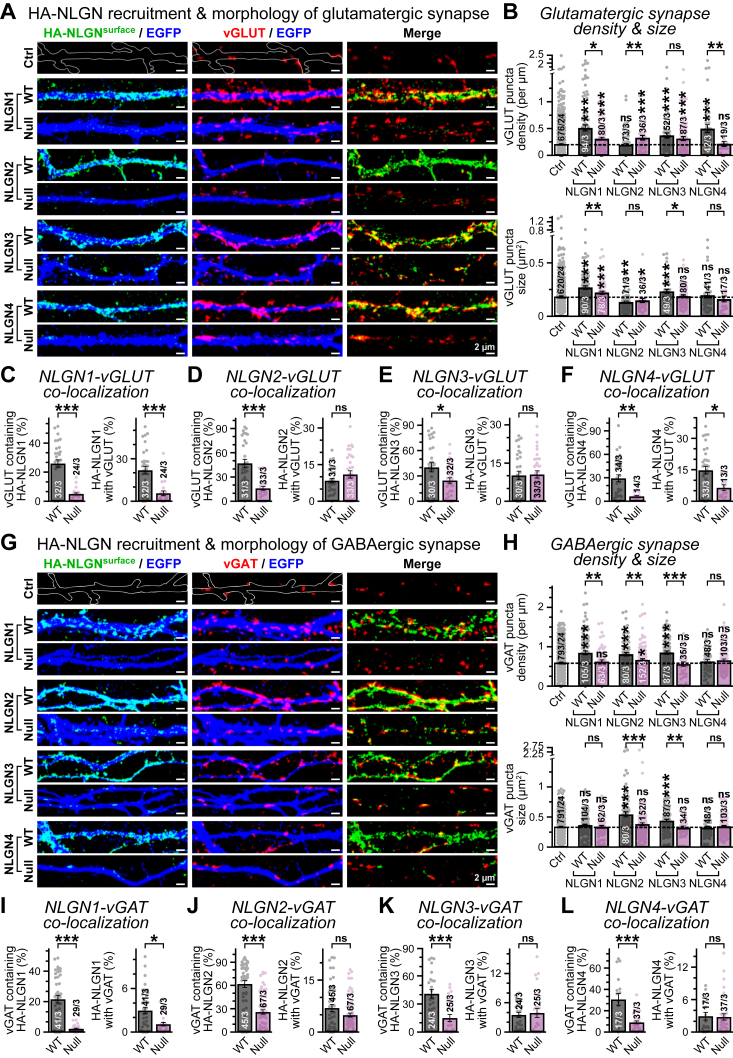
**Glycosylation-deficient NLGNs exhibit limited association with synapses.** Primary hippocampal neurons lacking endogenous NLGN1/2/3/4 (Fig. 5*A*) were supplemented with WT *versus* glycosylation-deficient versions of HA-tagged NLGNs along with a diffused EGFP-reporter, immunolabeled for surface HA epitopes under nonpermeabilized conditions, subsequently permeabilized using Triton X-100, and then counterstained with either vGLUT (*A*–*F*) or vGAT (panels *G*–*L*)-specific primary antibodies. *A* and *B*, example images (*A*) demonstrate surface HA-NLGN clusters (*left panels*) for WT *versus* glycan-null variants of NLGN1–4 (*top* to *bottom*) and vGLUT-positive glutamatergic synapses (*middle panels*), as formed along the EGFP-labeled dendritic segments, or merged views of both HA and vGLUT channels (*right panels*); average quantifications (*B*) reflect glutamatergic synapse density (*top*) and size (*bottom*), for NLGN1–4 WT *versus* glycan mutants. Control panels (Ctrl, *A*) illustrate dendritic branches traced using diffused background vGLUT signals (*white outline*) and synaptic parameters (*B*) measured from untransfected neighboring neurons lacking NLGNs. *C*–*F*, average degrees of association between vGLUT-positive glutamatergic synapses and cell-surface puncta of HA-tagged overexpressed NLGN1 (*C*), NLGN2 (*D*), NLGN3 (*E*), and NLGN4 (*F*), for WT *versus* their respective glycosylation-deficient variants, as measured using Mander’s coefficients of colocalization between channels. *G* and *H*, same as *A* and *B*, except for vGAT-positive GABAergic synapses induced by NLGN variant overexpressions. I–*L*, same as *C*–*F*, except for colocalization between vGAT-positive GABAergic synapses and NLGN variants. All summary graphs represent means ± SEM, for number of images analyzed/experimental sets. Data points from individual samples are presented as *color-matched circles*. Statistical significances were assessed by nonparametric Mann–Whitney *U* test, with ∗∗∗*p* < 0.005; ∗∗*p* < 0.01; ∗*p* < 0.05; ns, *p* > 0.05. EGFP, enhanced GFP; HA, hemagglutinin; NLGN, neuroligin; ns, not significant.

We next assessed whether the loss of N-linked glycosylation can impact NLGN-dependent synaptic parameters. In hippocampal neurons lacking all endogenous NLGNs, we systematically overexpressed individual HA-tagged NLGNs (either WT or glycan-deficient versions, Fig. 5*A*), immunolabeled for surface HA epitopes under a nonpermeabilized condition, then permeabilized the cells, coimmunostained them with an antibody against the glutamatergic synaptic marker vGLUT, and monitored for possible effects on synapse morphologies (Fig. 6*A*).

In NLGN1/2/3/4^KO^ background, selective overexpression of individual NLGNs variably affected glutamatergic synapse features. All WT NLGNs, with the exception of NLGN2, significantly increased the recruitment of vGLUT clusters on dendritic branches compared with control group (Fig. 6, *A* and *B*). This phenotype was most prominent for NLGN1, which triggered a considerable rise in both density and size of vGLUT puncta; NLGN3 and NLGN4 also enhanced vGLUT density with minor to no changes in puncta size (Fig. 6*B*). All effects were substantially reduced for mutant NLGNs with respect to WT counterparts, confirming a typical loss-of-function phenomenon (Fig. 6*B*). The fraction of vGLUT puncta that contained HA-NLGN signals also consistently declined for all glycan mutants, which directly correlated with major impairments in their membrane trafficking and dendritic localization (Fig. 6, *C*–*F*). Furthermore, the percentage of dendritic NLGNs that successfully targeted to synapses was also diminished for some glycan mutants, especially for NLGN1 and NLGN4, suggesting that most remain in extrasynaptic regions (Fig. 6, *C*–*F*). Therefore, glycan-null NLGNs demonstrated a near-complete loss from glutamatergic synapses.

A similar trend was detected when we repeated the experiments to probe for GABAergic synapses (Fig. 6*G*). Coimmunostaining for surface HA-NLGNs and GABAergic synapse marker vGAT revealed notable increase in synapse number and size by WT NLGN2 and NLGN3 overexpressions in NLGN1/2/3/4^KO^ background, whereas NLGN1 only enhanced vGAT density without affecting the puncta size, and NLGN4 failed to induce any changes (Fig. 6, *G* and *H*). Once again, glycan-deleted NLGNs were unable to generate similar impacts on GABAergic synapse morphologies and produced little to no effects compared with control groups (Fig. 6, *G* and *H*). In addition, glycan-null NLGNs continued to display a limited colocalization also with vGAT puncta, confirming their minimal recruitment at GABAergic synapses (Fig. 6, *I*–*L*). Together, these data uncovered an indispensable role of N-glycosylation for all NLGN isoforms, which ensures their efficient dendritic distribution and functional contribution in synapse development.

## Discussion

Glycosylation of several neuronal proteins has been reported to influence crucial biological programs, for example, neurite extension and axonal guidance (75, 76, 77), membrane excitability and ion-channel activity (78, 79), neurotransmitter release and reception (80, 81, 82, 83). However, the impact of glycosylation on synapse assembly and maintenance that are primarily mediated by synaptic adhesion complexes remains unclear. Here, we conducted a comprehensive and systematic functional assessment of multiple N-glycosylation sites in NLGN family proteins, a major class of synaptic CAMs. Our findings allow a number of principal conclusions about the role of glycosylation in regulating NLGN properties, and more broadly, emphasize on their mechanistic significance in synapse organization.

We observed that endogenous NLGNs are heavily glycosylated in mouse brain (Fig. 1, *A*, *B*, *E*, and *F*). Interestingly, for NLGN1 but not NLGN2, the extent of glycosylation was found to be distinct in cortical and hippocampal areas compared with several other brain regions, suggesting uneven level or composition of their glycan content (Fig. 1, *I* and *J*). These variabilities could potentially originate from differences in glycosylation pathways across multiple brain areas, as reported previously (84, 85). NLGN glycosylation patterns, however, were found to be similar between male and female sexes (Fig. 1, *A* , *B*, *E*, and *F*). Thus, the extent of NLGN glycosylation varied in a brain region–specific and isoform-dependent manner but irrespective of gender. A similar regional diversity in N-glycosylation profile was seen in SynCAM family, another prominent CAM in brain (86, 87, 88). We also detected substantial glycosylation in both NLGN2 and NLGN4X in human neurons, confirming that this PTM is species independent (Fig. 1, *K*–*M*).

We noticed that removal of N-linked glycans from WT NLGNs by exposing them to PNGase F alone, although effectively reduced their MWs, but did not completely abolish the heavier products. A similar trend was observed for all NLGN isoforms, for both their native forms in neurons and overexpressed versions in HEK cells (Figs. 1, *A*, *B*, *E*, *F*, *L*, and *M* and S3). These data indicate that mature NLGNs are potentially processed by additional modifications that further increase their MWs. In support of this notion, we found that mature NLGN1–4 acquired considerable amounts of sialylation and O-linked glycosylation, which could only be cleaved by acute sialidase and O-glycosidase digestion (Figs. 1, *C*, *D*, *G*, *H*, *L*, and *M* and S3). The N-glycan mutants did not manifest subsequent sialylation or O-glycosylation, implying that these changes occur downstream of N-glycosylation (Fig. S3). Therefore, N-glycosylation can amplify the complexity of PTMs in NLGNs.

We described multiple consensus sequences for N-linked glycosylation within the AChE domains of NLGN1–4 (Fig. 3*A*). Depending on NLGN isoforms, alanine replacements of these individual asparagine residues exerted variable impacts on their MW, surface availability, or presynapse interaction, indicating that these sites could be differentially occupied by glycosylation (Figs. 3, *C*–*F* and S5). Of the two highly conserved N-glycosylation sequons, site I *versus* site V, the former generated more stringent phenotypes in most assays, implying its functional hierarchy in all NLGNs; while the unique positions II, III, and IV also influenced NLGN1 and NLGN2 properties. Although significant, the magnitudes of most single-site substitution phenotypes were relatively minor, especially with respect to their glycan-null versions (compare with Figs. 3*L* and 4, *H*–*K*), suggesting that individual sites are not uniformly modified but collectively define NLGNs’ ability to act as a *trans*-synaptic CAM.

N303 residue of NLGN1 site III is probably the best previously-characterized glycosylation sequon. However, it is located exclusively within splice site B and not conserved in any other NLGN subtypes (Fig. 3*A*). An N303A substitution at this site has been reported to enhance NLGN1’s binding affinity for NRXNs *in vitro* (45). Despite this, in a cellular environment, we noticed that N303A substitution also decreased proper maturation and cell-surface trafficking of NLGN1 (Figs. 3*C* and S5*A*). Overall, N303A substitution caused no net increase in synapse recruitment, possibly because of these two seemingly opposing effects (Fig. S5*E*). Hence, N-glycosylation is a crucial PTM for most NLGN residues, even at the expense of their reduced ligand affinities. Furthermore, most of the other glycosylation sites are situated distant from NLGNs’ Ca^2+^-binding interfaces (24, 89), and enzymatic removal of these additional polysaccharide chains or terminal sialic acid residues from NLGN1 or even complete deglycosylation of NRXN1β did not inhibit their association capacity (45). These findings imply that glycosylation may not directly modulate NLGNs’ molecular interaction with NRXNs, as a general property.

N-glycosylation is required for proper folding and/or stability of several transmembrane proteins (67, 68, 69, 70, 71, 72). We probed for any such impacts on NLGNs by measuring their thermo-aggregation propensities as well as their protection from proteolytic cleavages, two conventional methods that have previously been utilized to estimate any sizable impact on overall protein structure (71, 90, 91, 92, 93, 94). However, compared with WT, glycan-null NLGN1–4 exhibited no significant differences in their thermal denaturation (Fig. 3, *G* and *H*). We did observe a significant but small decrease in protease susceptibility for the null mutants, which could reflect either a minor effect on folding, or reduced steric hindrance in protease accessibility because of loss of glycan side chains as reported before (95, 96, 97, 98). Removal of N-glycosylation also did not significantly alter the total protein levels of NLGN1–4 (Fig. S4), implying no immediate degradation *via* unfolded protein response (99, 100, 101). Furthermore, although the cell-surface localization of glycan-deleted NLGN1–4 was substantially low with respect to WT versions, they largely preserved their ability to interact with NRXNs and recruit presynaptic terminals, especially when compared with control condition (Fig. 4). Moreover, all glycosylation residues were located away from NLGNs’ dimerization domains and transmembrane helices (Fig. S3). Thus, loss of glycosylation does not apparently cause any major conformational destabilization of NLGN isoforms, at least in our *in vitro* and *in vivo* assays. However, we cannot completely rule out potential effects of aggregation in a complex environment influencing NLGNs’ affinity for binding partners.

Loss of N-linked glycosylation primarily impaired NLGNs’ proper trafficking to the cell surface. This phenotype was similarly observed in neurons and HEK cells, indicating a cell-autonomous mechanism that directly affected NLGNs’ molecular properties (Figs. 3, *K* and *L*, 5, *C*–*F*, and S5). In both cases, the glycan-deficient NLGN1–4 were primarily retained within intracellular secretory compartments, mostly at the ER (Figs. 3, *M*–*O* and 5, *G*–*L*). We found that NLGN variants are modified by multiple glycan subtypes, including high-mannose, hybrid, and complex forms (Figs. 2, *C*–*F* and S1). Interestingly, the ER-associated and predominantly high-mannose containing core N-linked glycans but not their subsequent maturation into hybrid or complex forms in the Golgi apparatus was necessary for NLGNs’ surface transport (Fig. 2, *G*–*J*). These results suggest that NLGNs can follow an unconventional secretory processing, similar to several other neuronal surface proteins (102).

Glycosylation defect in neuronal proteins has been linked to severe psychiatric diseases (103, 104, 105). Here, we found that elimination of N-glycosylation residues can markedly reduce NLGNs’ availability at both glutamatergic and GABAergic synapses, triggering a major loss in their CAM function (Figs. 4, 6, and S5). Of note, the HA-tagged NLGN variants exhibited relatively less stringency compared with endogenous NLGNs that preferentially localize at specific synapses in an isoform-dependent manner (4, 5, 6), likely because of their overexpression levels. Even then, the glycosylation-dependent surface delivery of HA-NLGNs essentially correlated with their postsynaptic recruitment. Notably, we and others have demonstrated that several disease-associated mutations in NLGN4X can also mechanistically affect its molecular properties *via* aberrant glycosylation, either directly or indirectly. For instance, an autism-related R101Q substitution neighboring the conserved N102 glycosylation site I can severely attenuate NLGN4X maturation, its cell-surface transport, and synaptic availability (20). Another autism mutation, that is, R87W in NLGN4X, also impaired its glycosylation profile and caused major loss in protein function, although this phenotype could be a downstream effect of protein misfolding (19). A cluster of additional NLGN4X mutations (*e.g.*, G84R, P94L, G99S, V109L) located adjacent to the critical N102 residue was also reported to increase its immaturely glycosylated fraction (25, 106), which could potentially allude to a common pathogenic mechanism.

## Experimental procedures

### Institutional approvals

All cell culture methods as well as lentivirus production procedures were authorized by the Institutional Biosafety Committee (protocol no.: #19-059B), Colorado State University. All experiments with mice were approved by the Institutional Animal Care and Use Committee (protocol nos.: #1412 and #1482).

### Mouse husbandry

For endogenous NLGN glycosylation (Fig. 1) and artificial synapse formation (Fig. 4) assays, WT C57BL/6J mice (Jackson Laboratory) were used. Generation and characterization of NLGN1/2/3^flox/flox^ triple cKO mice were described previously (10, 11, 12, 73, 74). In brief, exons of NLGN genes were flanked by LoxP sites and could be excised by acute expression of Cre-recombinase. These animals were further bred with constitutive NLGN4-like^KO^ mice (developed by Dr Nils Brose, Max Planck Institute) produced by earlier studies (107, 108) to obtain NLGN1/2/3/4^KO^ line (generously provided by Dr Thomas C. Südhof, Stanford University) for Figures 5 and 6.

### Mouse brain dissection

Adult (P60–P75) male and female WT mice of C57BL/6 strain were sacrificed, brains were extracted from decapitated heads, and placed into ice-cold Hank's balanced salt solution (HBSS) buffer. Different brain regions were surgically isolated under a dissection microscope (Olympus SZX16) immediately after. The tissues were cut into fine pieces and incubated in radioimmunoprecipitation assay (RIPA) buffer (150 mM NaCl, 5 mM EDTA, 25 mM Tris [pH 7.4], 1% Nonidet P-40 substitute, and 0.5% sodium deoxycholate) supplemented with Halt protease inhibitor cocktail (PIC; catalog no.: 78429; Thermo Fisher) for 6 to 8 h at 4 °C. The lysates were triturated, spun at 4000*g* for 5 min, supernatants with protein extracts were aliquoted, and stored at −20 °C prior to experiments.

### Primary hippocampal cultures

Primary neurons were derived from newly born (P0–P1) pups of NLGN1/2/3^f/f^4^KO^ (Figs. 5 and 6) or WT animals (Figs. 2 and 4). Dissected hippocampi were digested at 37 °C for ∼15 min inside a tissue-culture incubator, with 10 U/ml papain (catalog no.: LS003126; Worthington Biochem) in HBSS buffer containing 0.5 mM EGTA. The hippocampi were then washed thoroughly with HBSS, dissociated in Neurobasal Plus media (contains 1.8 mM CaCl_2_, Thermo Fisher) + B27 supplement (Thermo Fisher), additionally containing ∼10% fetal bovine serum (FBS; Atlas Biologicals) + 1% penicillin–streptomycin mix (Thermo Fisher). Triturated cells were seeded onto Matrigel (Corning, Sigma–Aldrich) precoated glass coverslips placed inside individual wells of 24-well dishes. The day of neuronal plating was considered as 0 days *in vitro* (DIV 0). During DIV 2 to 3, FBS concentration was gradually reduced to ∼5% and then to ∼2.5% by adding almost equal volumes of Neurobasal media without any serum and included 5-fluorodeoxyuridine (10 μM) to prevent glial proliferation after reaching 70 to 80% confluency. These cultures were subsequently infected with lentiviruses expressing Cre-recombinase and transfected with NLGN WT or glycosylation-deficient constructs (see below). Fresh media of 50% volume were added at DIV 10.

### Cell lines

The HEK 293T (containing SV40 T-antigen) cells were commercially available from Takara Bio USA (catalog no.: 632180). The human iPS cells carrying doxycycline-inducible Ngn2 transgene (*i.e.*, the WTC-11 line, (63)) was a gift from Dr Michael E. Ward, National Institute of Neurological Disorders and Stroke.

### Generation of human neurons

The WTC-11 cells were maintained in mTeSR1 or mTeSR Plus media (StemCell Technologies) under feeder-free condition. Media were changed every day. When cell density reached 60 to 70%, they were dissociated using PBS + 0.5 mM EDTA and replated at ∼1:6 dilution onto Matrigel-coated wells. During passage, these cultures were in addition supplemented with ROCK-inhibitor Y-27632 (2.5 μM; MedChem Express) overnight but excluded from later media changes. For neural differentiation, these cells were plated at 1:15 dilution, induced by a continuous doxycycline (2 μg/ml) exposure, and cultured with N3 media (compositions: Dulbecco's modified Eagle's medium/nutrient mixture F-12 [DMEM/F12, Thermo Fisher] + insulin [20 μg/ml; Sigma–Aldrich] + N2 and B27 supplements [Thermo Fisher] + 1% penicillin/streptomycin). Cells were harvested 5 to 6 days after reprogramming was initiated, that is, when they already suppressed pluripotent identity and committed to neural fate (Fig. S2, (109)).

### Vector design

Reliable antibodies against NLGN extracellular domains do not exist. Hence, we introduced an HA epitope tag in NLGN coding sequences after their corresponding N-terminal signal peptide (rat NLGN1: 1–45, rat NLGN2: 1–14, mouse NLGN3: 1–34, and human NLGN4X: 1–43 amino acids) cleavage sites, which does not interfere with their cell-surface trafficking (10, 35, 62). Alanine substitutions for individual and all (referred to as “null”) potential N-linked glycosylation sites within the AChE domains of NLGN coding sequence were performed using site-directed mutagenesis. These constructs were inserted into a vector preceded by cytomegalovirus promoter, a Kozak sequence, and followed by an EGFP cassette driven by internal ribosome entry site (Fig. 3*B*). The NRXN1β construct without splice site 4 (NRXN1β^-S4^) was cloned from rat complementary DNA preparation and coexpressed with internal ribosome entry site-mOrange reporter (Fig. 4*A*).

### Lentivirus production

Three helper plasmids (*i.e.*, pRSV-REV, pMDLg/pRRE, and VSV-G; 7.5–8 μg each) and an expression vector encoding Cre-recombinase (15–20 μg; fused with EGFP, containing a nuclear localization signal, and driven by synapsin-1 promoter; see Ref. (110)) were cotransfected with polyethylenimine into 70 to 80% confluent HEK 293T cells plated on 10 cm dishes. Around 10 to 12 h post-transfection, the culture medium was exchanged completely, and the supernatant containing virus particles was collected after 36 and 60 h. The supernatant was then pooled and spun at ∼800*g* for 6 to 8 min to remove any HEK cell debris. The supernatant was then spun at ∼120,000*g* for 2 h at 4 °C (using a Beckman L8-70M ultracentrifuge, equipped with SW41Ti rotor). The lentiviral pellets were resuspended in ∼100 μl DMEM, stored overnight at 4 °C, subsequently aliquoted, frozen, and stored at −80 °C prior to experimental usage.

### NLGN overexpression

HEK cells were transfected with NLGN constructs using polyethylenimine, media were exchanged at 8 and 24 h, and cells were analyzed between 48 and 72 h by immunocytochemistry and Western blots. For primary neurons, DIV 3 hippocampal cultures derived from NLGN1/2/3^f/f^4^KO^ animals were first infected with Cre-EGFP lentiviruses. On DIV 8 to 10, neurons were in addition transfected with NLGN WT *versus* glycan-null mutants using Lipofectamine 3000 (Thermo Fisher) reagent and immunostained 6 days afterward, that is, at DIV 14 to 16 (Fig. 5*A*).

### Cell-aggregation assay

Separate HEK cell cultures were transfected with either NRXN1β-mOrange or different NLGN constructs with EGFP reporter. An EGFP-only vector was used as negative control. After 48 h, cells were washed with PBS and lifted with 1 mM EDTA. They were spun at ∼800*g* for 5 min, the pellets were triturated and resuspended at a density of 1 × 10^6^ cells/ml in DMEM (Genesee Scientific) supplemented with 10 mM CaCl_2_. Equal amounts of NRXN- and NLGN-expressing cells were mixed together (100 μl each) at 37 °C for 2 h in a tube rotator. Mixtures were spotted onto glass slides with cut pipette tips and immediately imaged in tile-scan mode using an inverted STELLARIS 5 (Leica Microsystems) laser scanning microscope equipped with a 5× dry objective. Cell aggregates were analyzed using Mander’s colocalization coefficient.

### Artificial synapse formation

Primary hippocampal neurons containing limited number of astroglia cells were obtained from E16.5 embryos of timed-pregnant WT C57BL/6J mice and seeded onto poly-d-lysine (catalog no.: P0899; Sigma–Aldrich)-coated glass coverslips, at a density of 100 to 200 cells/mm^2^. Neurons were cultured in Neurobasal media (supplemented with FBS (Hyclone), GlutaMAX (1:400; Thermo Fisher), penicillin/streptomycin, N21-MAX (1:50; R&D Systems), and 5-fluorodeoxyuridine; the media were half-exchanged (50% fresh + 50% glia-conditioned media) every 2 days. At DIV 10 of the neuronal cultures, separately maintained HEK cells were transfected with respective NLGN constructs or an EGFP-only negative control. After 48 h, these HEK cells were lifted with PBS + EDTA, pelleted down, triturated as single cells, and then resuspended in Neurobasal medium. Transfected HEK cells were counted using EGFP fluorescence and distributed at a density of ∼3000 cells into each well containing DIV 12 neurons. After 4 days of coculture, coverslips were fixed with 4% paraformaldehyde, dissolved in PBS, and stained as described later.

### Thermal stability and protease sensitivity assays

For thermal aggregation tests (Fig. 3, *G* and *H*), the lysates of HEK cells expressing NLGN1–4 WT *versus* corresponding glycan-null versions for ≥48 h were collected in RIPA buffer + PIC, heated for 15 min at different temperature gradients in a thermocycler (Bio-Rad T100). The samples were then centrifuged at ∼15,000*g* for 5 min in order to precipitate aggregated protein fractions, the supernatants were collected, and analyzed using Western blots.

For protease degradation tests (Fig. 3, *I* and *J*), the transfected HEK cells were lysed and collected in RIPA buffer as described but without any PIC. Immediately after collection, crude lysates were distributed into individual tubes and treated with varying concentrations of trypsin (catalog no.: PR-V5280; Fisher Scientific). After 5 min, reactions were quenched by adding 10% SDS loading buffer and analyzed by Western blots.

The data (*i.e.*, band intensities) from thermal-stability experiments or protease sensitivity assays were first normalized to their corresponding starting samples, that is, respectively, lowest incubation temperature (37 °C, for thermal-stability assay; Fig. 3*G*) or protease-free condition (0 μM trypsin, for protease sensitivity assay; Fig. 3*I*), then fitted with Boltzmann sigmoidal curves $\left(y=\frac{1}{{1+e}^{\frac{V50-x}{Slope}}}\right)$ to compute V50 values (*i.e.*, 50% of the peak) for each experimental trial, and compared between NLGN1–4 WT *versus* glycan-null groups from multiple batches.

### Glycosidase treatment

For glycosidase treatment of endogenous NLGNs, 20 μl lysates from different mouse brain regions or differentiated human neurons were denatured at 70 °C (to minimize NLGN aggregation) and treated with either α2-3,6,8,9 neuraminidase A (sialidase; catalog no.: P0722; New England Biolabs) or PNGase F (catalog no.: P0704; New England Biolabs) for 3 h at 37 °C, according to the manufacturer's specifications. The control samples were treated similarly but with equal volumes of RIPA buffer instead of glycosidases.

For glycosidase treatments of HA-tagged NLGNs overexpressed in HEK cells, lysates were incubated with mouse anti-HA antibody (1:1000 dilution; catalog no.: h3663; Sigma–Aldrich) in a tube rotator overnight at 4 °C. The lysates and antibody mix were incubated with protein G-Sepharose beads (catalog no.: 6511; BioVision) while rotating for 3 to 4 h at 4 °C. The beads were washed three times with RIPA buffer + PIC, aliquoted into four equivalent fractions, centrifuged at ∼15,000*g* for 4 min, and then resuspended into 20 μl RIPA buffer. Aliquots were exposed to either PNGase F, or sialidase, or PNGase F + sialidase + O-glycosidase (catalog no.: P0733S; New England Biolabs), per the manufacturer specifications for 3 h at 37 °C in a tube rotator. To elute deglycosylated NLGNs, the beads were centrifuged, supernatants were removed, and 15 μl SDS loading buffer was added. Samples were boiled for 5 to 10 min, centrifuged at ∼15,000*g* for 4 min, and eluates were collected for immunoblotting.

### Glycosylation inhibitor treatments

Human neurons derived from WTC-11 iPS cells were incubated with drugs (*i.e.*, Tm [0.25 and 0.5 μM], Kf [5 μM], Dmj [75 μM], or Sw [100 μM]) dissolved in N3 media, starting at day 4 postdifferentiation. Equal volumes of dimethyl sulfoxide were used as control treatment. Cells were lysed after 48 h, protein extracts were collected, and subsequently analyzed by quantitative immunoblots.

### Surface biotinylation

Neuronal surface proteins were labeled with biotin by Pierce Cell Surface Biotinylation and Isolation Kit (catalog no.: A44390; Thermo Fisher) following the manufacturer’s specifications. Briefly, cells were incubated in PBS containing sulfo-NHS-SS-biotin and biotin ligase for 10 min at room temperature, then washed with Tris-buffered saline (TBS) to quench the reaction. Neurons were then lifted and pelleted, and whole-cell lysates were extracted and incubated with NeutrAvidin agarose beads to isolate biotin-labeled proteins. As the NeutrAvidin agarose beads were washed, the initial flow-through was saved and used as intracellular protein fraction. After beads were washed and biotinylated proteins eluted, internal and surface fractions were diluted as necessary.

### Lectin-based precipitation

Protein extracts (whole-cell lysate, surface *versus* intracellular fraction) were mixed with agarose beads bound with following lectins: ConA (catalog no.: AL-1003; Vector Labs), RCA (catalog no.: AL-1083; Vector Labs), or WGA (catalog no.: AL-1023; Vector Labs). The bead–lysate mixtures were incubated overnight at 4 °C while rotating end over end. Beads were pelleted by light centrifugation and rinsed three times with ice-cold PBS. To release glycoproteins bound by the lectin-agarose beads, pellets were resuspended in 20 to 40 μl SDS loading buffer and boiled for 10 min. The mixtures were vortexed, centrifuged at 16,000*g* for 3 min, and eluates were collected to run on SDS-PAGE. Agarose beads without lectins were used as a negative control.

### Immunoblotting

Transfected HEK cells were lifted with 1 mM EDTA in PBS and lysed in RIPA buffer with PIC. Alternatively, samples were collected from various mouse brain regions or iPS cell (WTC-11)–derived human neurons. The protein extracts were mixed with 10% SDS loading buffer, then run on either 5%, 7.5%, or 10% SDS-PAGE, and transferred to nitrocellulose membrane. The membranes were blocked with 5% bovine serum albumin in TBS containing 0.1% Tween-20 detergent (TBS-T) while rocking for at least 1 h at 37 °C, and then incubated overnight at 4 °C with corresponding primary antibodies diluted in blocking buffer. Primary antibodies included mouse anti-NLGN1 (1:500 dilution; catalog no.: 129111; Synaptic Systems) or rabbit anti-NLGN2 (1:1000 dilution; catalog no.: 129202; Synaptic Systems), or rabbit anti-NLGN4X (1:1000 dilution; catalog no.: A7986; ABclonal), or rabbit anti-NLGN4Y (1:1000 dilution; catalog no.: A4513; ABclonal), or mouse anti-HA (1:1000 dilution; catalog no.: h3663; Sigma–Aldrich), or chicken anti-GFP (1:500 dilution; catalog no.: GFP-1020; Aves Labs), or mouse anti-GAPDH (1:50,000 dilution; catalog no.: 60004-1-Ig; Proteintech). The blots were thoroughly washed for five times with TBS-T over an hour, subsequently incubated with fluorescent dye–conjugated secondary antibodies (1:2000 dilution; DyLight 680/800; Invitrogen) diluted in blocking buffer (TBS-T, containing bovine serum albumin) for 1 to 2 h, washed again for five times with TBS-T, and then imaged immediately using a LI-COR Odyssey CLx system.

The brightness and contrast of all protein bands were uniformly adjusted using Image Studio Lite (version 5.2, LI-COR Biosciences) software. Protein contents were estimated by plotting total-intensity profiles for each lane and calculating the peak areas underneath. For glycosidase assays, the Y-coordinates of individual peaks were recorded, and mobility shifts were normalized to the visible dye front as well as a 100 kDa ladder (catalog no.: P007; GoldBio). Of note, the NLGN1 and NLGN2 bands from mouse brain lysates, both before and after glycosidase treatment, were confirmed for minimal crossreactivity (Fig. S1*A*); whereas, the NLGN2- and NLGN4X-specific bands were also validated using extracts from human neuron KO for individual genes (data not shown).

### Immunostaining

Primary mouse neurons, differentiated human neurons, and HEK cells cultured on coverslips were washed once with PBS and fixed immediately with 4% paraformaldehyde for 30 min at room temperature, followed by an additional PBS wash. Cells were blocked in PBS + 10% cosmic calf serum (HyClone) for at least 1 h at 37 °C while rocking, incubated with primary antibodies for 2 h at 37 °C or overnight at 4 °C, washed four times with blocking buffer, and incubated with Alexa Fluor 488/555/647 dye (Invitrogen)-conjugated secondary antibodies for 1 to 2 h at 37 °C. Coverslips were then washed twice with blocking buffer and twice with PBS and mounted upside down on glass slides using Fluoromount-G (Southern Biotech). To detect HA epitopes on NLGNs localized at the cell surface, cultures were stained under nonpermeabilized conditions, that is, without any Triton X-100. For assays where permeabilization was required, Triton X-100 (0.1%) was subsequently applied to blocking buffer and for all following steps, including washes as well as primary or secondary antibody dilutions.

Primary antibody combinations included mouse anti-Oct3/4 (1:500–1000 dilution; catalog no.: sc-5279; Santa Cruz Biotechnology), mouse anti-Ki-67 (1:500 dilution; catalog no.: 550609; BD Biosciences), mouse anti-Tuj1 or β-tubulin III (1:500 dilution; catalog no.: 801202; BioLegend), chicken anti-MAP2 (1:500 dilution; catalog no.: ab5392; Abcam), rabbit anti-Synapsin1/2 (1:1000 dilution; catalog no.: 106002; Synaptic Systems), mouse anti-HA (1:1000 dilution; catalog no.: h3663, Sigma–Aldrich), chicken anti-GFP (1:500 dilution; catalog no.: GFP-1020; Aves Labs), rabbit anticalnexin (1:500 dilution; catalog no.: PA5-34754; Thermo Fisher), rabbit anti-GM130 (1:300 dilution; catalogno.: A5344; ABclonal), rabbit anti-vGluT1 (1:500 dilution; catalog no.: 135303; Synaptic Systems), rabbit anti-vGAT (1:500 dilution; catalog no.: 131003; Synaptic Systems). The cell nuclei were stained with 4′,6-diamidino-2-phenylindole (1:50,000 dilution; catalog no.: D1306; Thermo Fisher) for 5 to 10 min in washing buffer.

### Image acquisition and analysis

Images were captured using STELLARIS 5 confocal microscope equipped with Power HyD detectors and LasX software (Leica Microsystems). Series of optical z-sections (∼0.5–1 μm thickness) were acquired with 20× (dry, 0.75 numerical aperture [NA]), 40× (oil immersion, 1.3 NA), and 63× (oil immersion, 1.4 NA) plan-apochromatic objectives.

Images were processed and analyzed using FIJI-ImageJ (National Institutes of Health) software. Surface trafficking of HA-tagged NLGNs expressed in HEK cells or neurons was monitored by maximum intensity z-projections of HA channels and normalized to corresponding EGFP signals. Subcellular distribution of HA-NLGNs (Fig. 3*N*) was assessed by measuring signal intensities across line profiles and normalizing them to maximum binned values. In order to quantify synaptic specifications (*e.g.*, puncta density, size, total intensity) formed along neuronal processes, z-projected synaptic signals from regions of interest were superimposed and normalized with respect to the length of corresponding EGFP-labeled dendritic segments (Figs. 5 and 6). For artificial synapse formation assays (Figs. 4, *G*–*K* and S5), the synaptic parameters were similarly normalized by HEK cell surface area. Colocalizations between two markers (*e.g.*, HA-NLGNs with CNX, GM130, vGLUT, vGAT) were assessed by first thresholding individual channels appropriately to eliminate background signals for different experimental batches and then measuring Mander’s coefficients for each optical section within the JACoP plugin.

### Structural prediction of NLGNs

The Protein Data Bank files containing full-length amino-acid sequences of rat NLGN1, rat NLGN2, mouse NLGN3, and human NLGN4X were downloaded from AlphaFold (protein structure database) web portal. These resulting Protein Data Bank files were then modeled individually, aligned with top views of their dimerization helices, and colored using ChimeraX software (University of California, San Francisco). Ribbon models with secondary structures were chosen to visualize and annotate the N-linked glycosylation sites (Fig. S3, *A*–*D*).

### Data presentation

For all experiments, all average values indicate means ± SEM (SD of a parameter divided by square root of number of samples) and are presented along with number of independent observations, for example, number of animals (immunoblots: mouse brain samples) or cultures (immunoblots: HEK cells and human neurons) examined, or field of views analyzed/number of experimental batches (immunostaining: HEK cells and mouse primary neurons). For near-normally distributed datasets, statistical evaluations between conditions were conducted using unpaired (paired for batch-wise comparisons), two-tailed, Student’s *t* test (∗∗∗*p* < 0.005; ∗∗*p* < 0.01; ∗*p* < 0.05; ns = not significant, *p* > 0.05); nonparametric Mann–Whitney *U* test was used otherwise. Single- or two-factor ANOVA was performed for groupwise assessments, as mentioned.

## Data availability

Individual data points are provided in all the figures (both main and supporting information) along with average quantifications. The raw data and experimental reagents are available upon request to S. C. (soham.chanda@colostate.edu).

## Supporting information

This article contains supporting information (Supplementary Figures S1–S5 with figure legends).

## Conflict of interest

The authors declare that they have no conflicts of interest with the contents of this article.
