# Supplementary material for: Multiple N-linked glycosylation sites critically modulate the synaptic abundance of neuroligin isoforms
Source: J Biol Chem. 2023 Oct 20;299(12):105361. doi: 10.1016/j.jbc.2023.105361 (PMC10679506; doi:10.1016/j.jbc.2023.105361)
Supplement: Supplemental figures and legends [file mmc1.pdf]

Supplementary Figures & Figure Legends

Multiple N-linked glycosylation sites critically modulate the synaptic abundance of Neuroligin isoforms

Orion Benner[1,*], Thomas P. Cast[1,*], Laurie S. Minamide[1], Zephyr Lenninger[2], James R. Bamburg[1,2,3], and Soham Chanda[1,2,3,#]

[1]Biochemistry & Molecular Biology, Colorado State University, Fort Collins, CO 80523, USA;
[2]Molecular, Cellular & Integrated Neurosciences, Colorado State University, Fort Collins, CO 80523, USA;
[3]Cell & Molecular Biology, Colorado State University, Fort Collins, CO 80523, USA

*Co-first author
[#]Correspondence & Lead Contact: SC (soham.chanda@colostate.edu)

List of Supplementary Figures:

Supplementary Figure S1 (Related to Figure 1, Figure 2, and Figure 3)**:**
Major NLGN isoforms are heavily modified by diverse N-glycan subtypes.

Supplementary Figure S2 (Related to Figure 1 and Figure 2)**:**
WTC-11 iPS cells suppress pluripotency and rapidly reprogram into neurons.

Supplementary Figure S3 (Related to Figure 3)**:**
WT NLGNs but not the glycan-null variants undergo extensive glycosylation.

Supplementary Figure S4 (Related to Figure 3 and Figure 4)**:**
Lack of glycosylation impairs NLGNs' surface export via secretory pathway.

Supplementary Figure S5 (Related to Figure 5 and Figure 6)**:**
Glycan mutations impair NLGN-mediated assembly of presynaptic terminals.

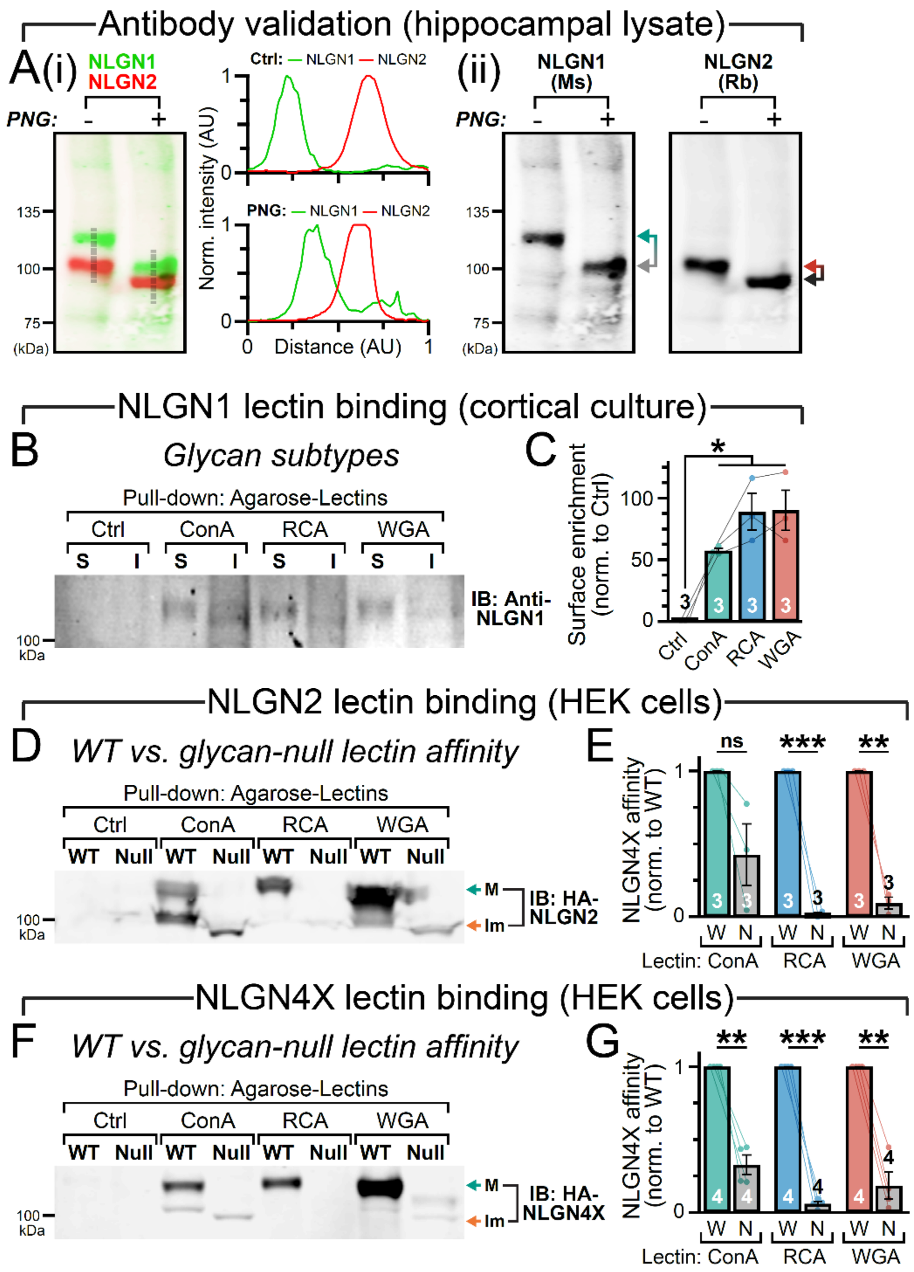

<u>Supplementary Figure S1:</u> **Major NLGN isoforms are heavily modified by diverse N-glycan subtypes.**

A. Example image (**i**, left) and band intensity profile (**i**, right; along the dotted lines) of a western-blot from mouse hippocampal sections, probed simultaneously with both NLGN1 (mouse = Ms) and NLGN2 (rabbit = Rb) primary antibodies, and labeled using fluorophore-conjugated (700 vs. 800 nm) anti-Ms vs. anti-Rb secondary antibodies; split views of respective channels (**ii**), without (Ctrl, "-" symbol) and with (PNG, "+" symbol) PNGase F treatments. The connected arrowheads (in panel **ii**) indicate distinct banding patterns for NLGN1 (green channel) vs. NLGN2 (red channel) proteins, and a noticeably different mobility shift in their corresponding MWs following the PNGase F glycosidase digestion. AU = Arbitrary Unit. Note that all data in Fig. **1A-J** were generated similarly.

B-C. Sample western-blot (**B**) depicts surface (S) and internal (I) fractions of endogenous NLGN1 expressed in mouse cortical cultures, following surface biotinylation and precipitation with lectin-conjugated agarose beads to ascertain their respective glycosylation pattern (see Fig. **2B**); summary plot (**C**) of surface NLGN1 lectin affinity.

D-E. Example immunoblot (**D**) and summary graph (**E**) of WT vs. glycan-null NLGN2 overexpressed in HEK 293 cells, and pulled down from whole cell lysates using ConA, RCA, or WGA -bound agarose beads (as indicated).

F-G. Same as panels **D-E**, except for HEK 293 cells that were transfected with human NLGN4X.

All quantifications represent averages (means ± SEM), with total number of independent batches (insets in the bar-graphs) and individual data-points (color-coded connected circles). All statistical analyses were conducted by one-tailed, paired, Student's t-test, with *** $P < 0.005$; ** $P < 0.01$; * $P < 0.05$; ns = not significant, $P > 0.05$.

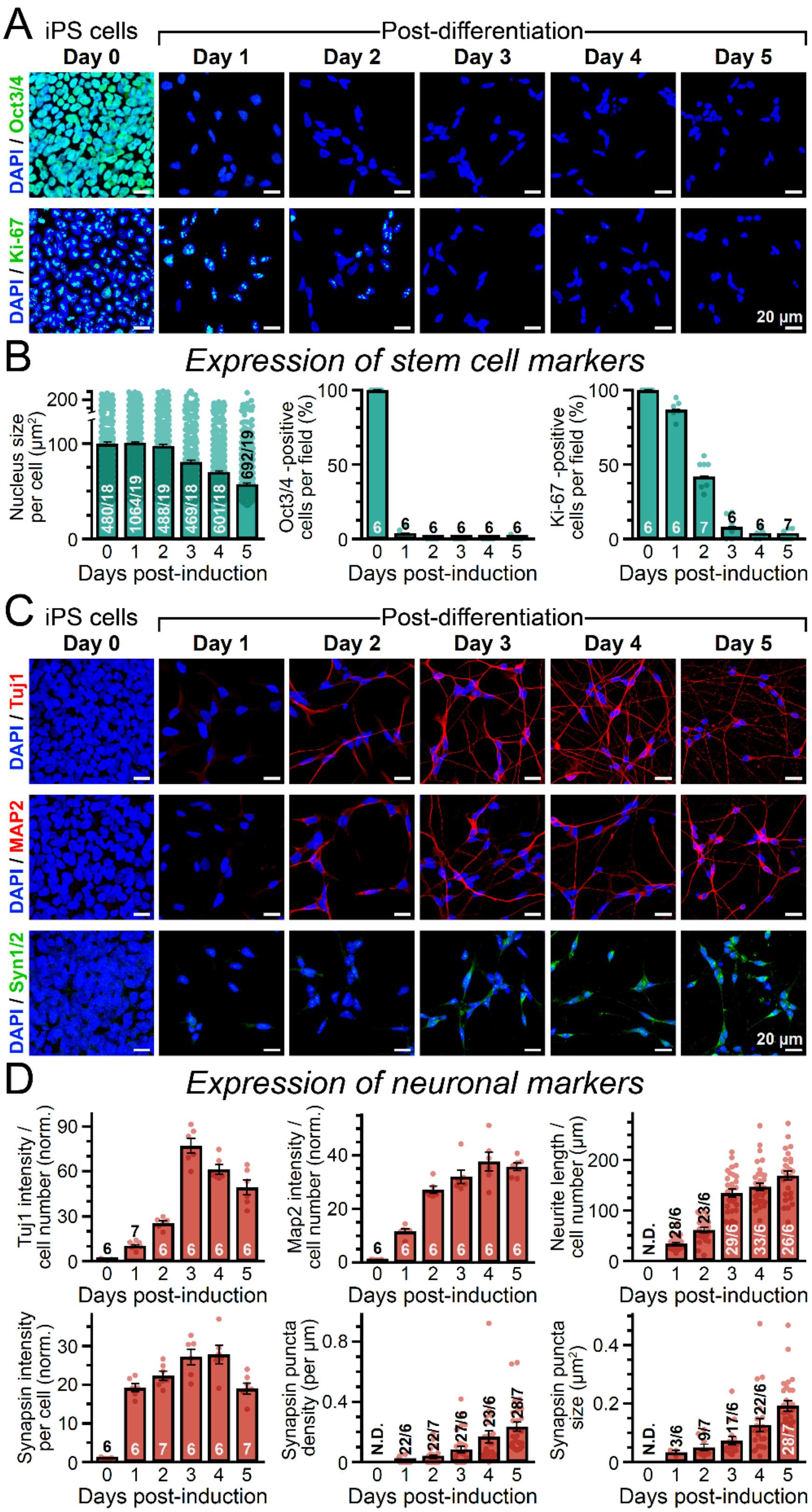

Supplementary Figure S2: **WTC-11 iPS cells suppress pluripotency and rapidly reprogram into neurons.** Human iPS cells (i.e., WTC-11 line) were directly reprogrammed into neurons by doxycycline-induced Ngn2 overexpression (Day 0), and immunostained subsequently (Day 1-5) with appropriate markers, as indicated.

A-B. Sample images (**A**) of cells labeled for stem cell marker Oct3/4 or proliferation marker Ki-67, and nuclear stained with DAPI; summary plots (**B**) of nuclei size and respective fractions of Oct3/4 or Ki-67 -positive nuclei, as calculated by setting the Day 0 minimal intensity values as threshold cut-offs for corresponding signals.

C-D. Representative field-of-views (**C**) of cells stained with nuclear DAPI, co-labeled for pan-neuronal markers Tuj1, Map2, or Synapsin; average values of Tuj1 and Map2 signal intensity with total neurite length (**D**, top), or intensity of Synapsin signals distributed along newly formed neurites, with puncta density and size (**D**, bottom).

All summary graphs are plotted as means ± SEM, and provided with number of cells analyzed / field-of-views, or only the total number of field-of-views. All individual data-points are included as color-matched filled circles. Results demonstrate an accelerated transition of the human iPS cells from a pluripotent stage towards mature neuronal identity (from post-induction Day 0 to Day 5), with robust changes in their relevant marker expression.

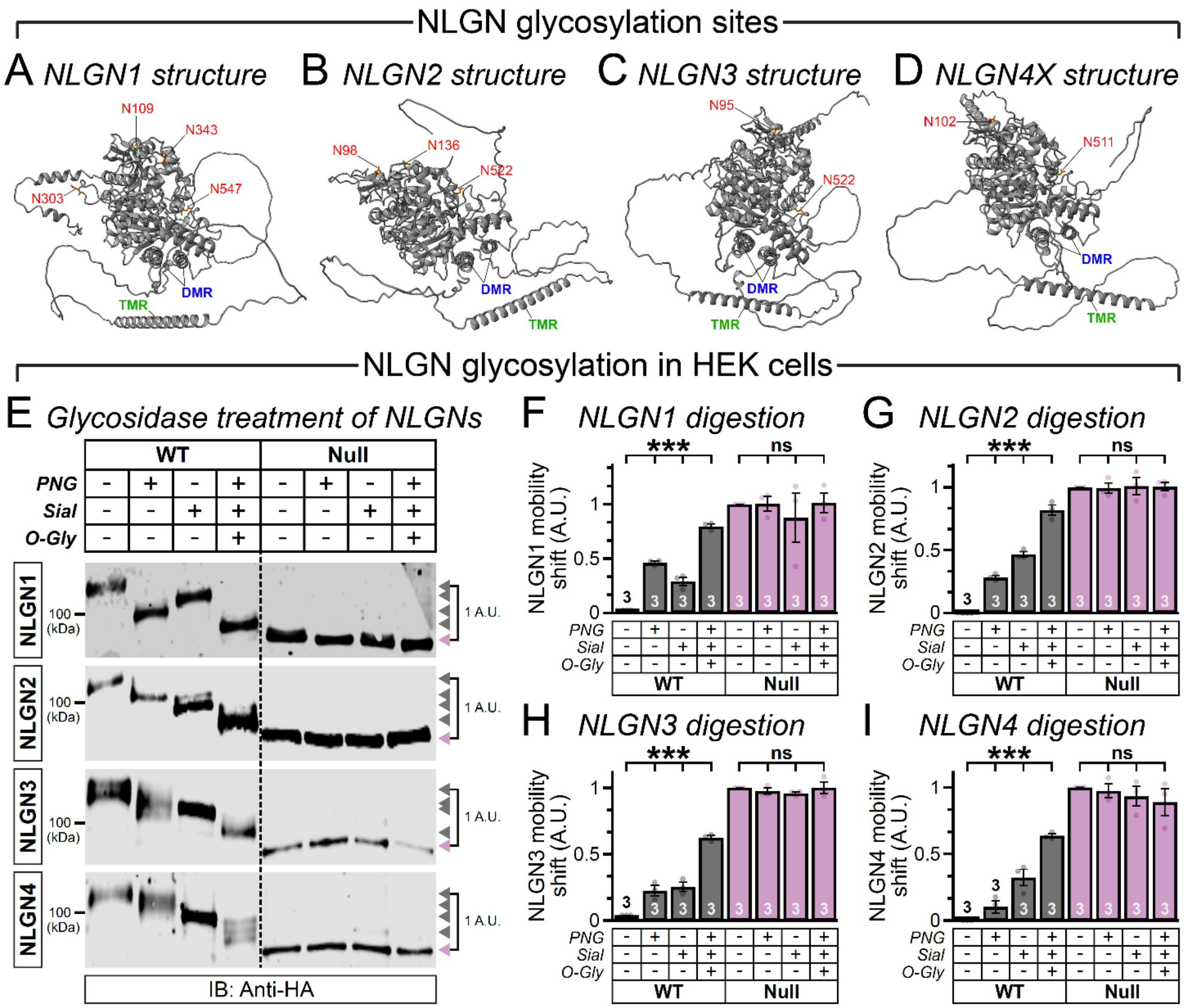

Supplementary Figure S3: **WT NLGNs but not the glycan-null variants undergo extensive glycosylation.**

A-D. Structural models of NLGN1 (**A**), NLGN2 (**B**), NLGN3 (**C**), and NLGN4X (**D**) indicating spatial orientations of the potential N-linked glycosylation sites, away from dimerization (DMR) and transmembrane (TMR) regions.

E-I. HEK cells were transfected with HA-tagged WT NLGNs (NLGN1-4) vs. their corresponding Null variants; protein extracts were incubated with PNGase F (PNG), sialidase (Sial), or O-glycosidase (O-Gly), as indicated ('+' treated, '-' untreated), and the mobility shifts of individual NLGN bands were probed with anti-HA antibody. Representative immunoblots (**E**) of NLGN WT (left) vs. Null (right) variants, separated by a vertical dotted line. Changes in NLGNs' MWs after different glycosidase treatments were normalized by the total distance (1 A.U. = Arbitrary Unit) between each untreated WT (grey arrowheads) vs. glycan-null (purple arrowheads) versions. Relative migration of NLGN1 (**F**), NLGN2 (**G**), NLGN3 (**H**), and NLGN4 (**I**) bands, after glycosidase digestions.

All quantifications represent means ± SEM; color-coded filled circles on the bar-graphs indicate individual data-points from independent experimental batches (n = 3, for all experimental conditions). Statistical comparisons between multiple groups were performed by one-way ANOVA, *** $P < 0.005$; ns = not significant ($P > 0.05$).

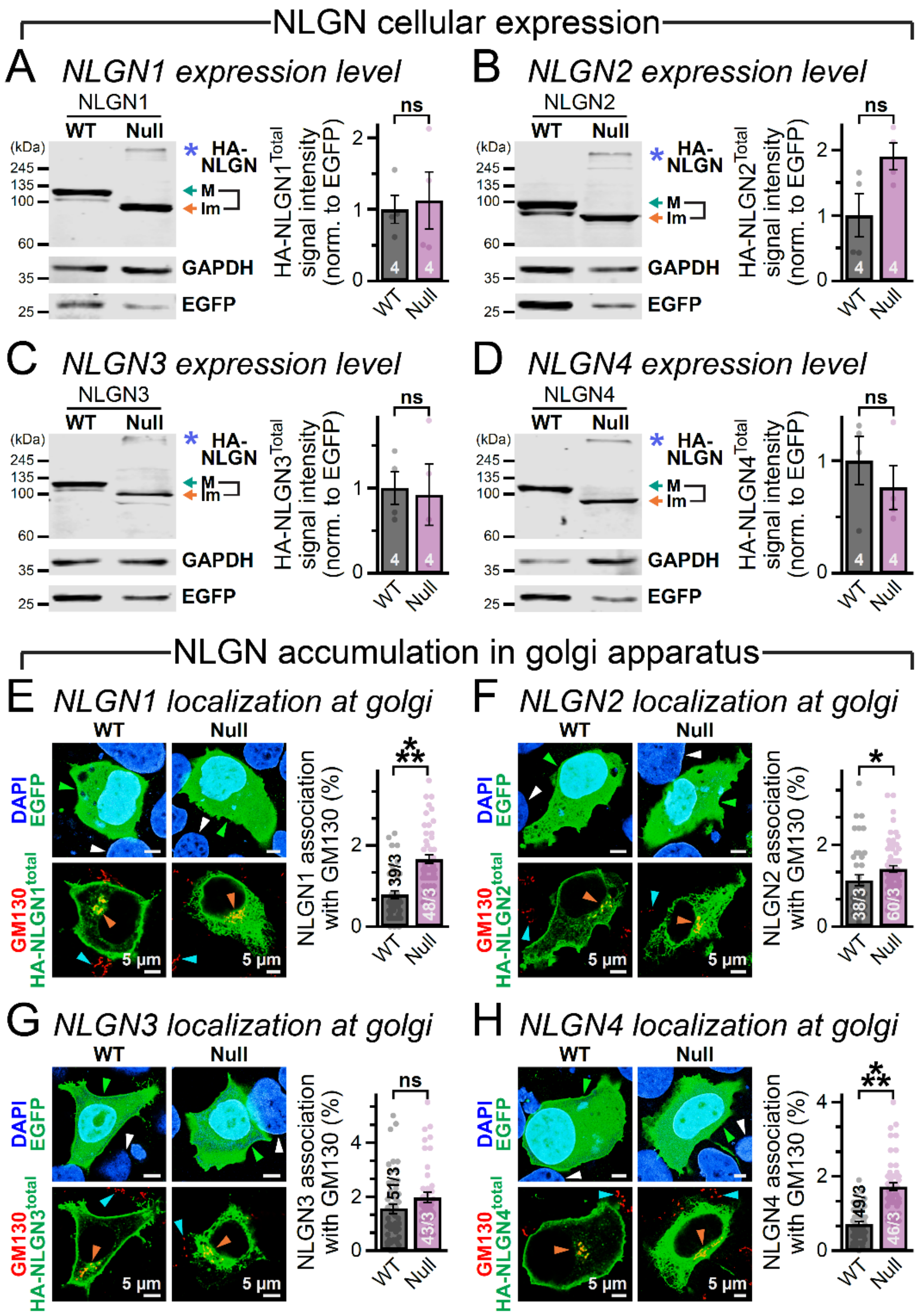

Supplementary Figure S4: **Lack of glycosylation impairs NLGNs' surface export via secretory pathway.**

HEK cells expressed HA-tagged WT vs. glycosylation-deficient NLGNs, followed by an IRES-EGFP cassette.

A-D. Western-blot samples (left) and summary plots (right) of overexpressed protein levels for WT vs. glycan-null versions of NLGN1 (**A**), NLGN2 (**B**), NLGN3 (**C**), and NLGN4 (**D**), when normalized to their corresponding EGFP signals. Arrowheads are maturely (M) vs. immaturely (Im) glycosylated NLGNs; asterisks = aggregated products frequently observed for all glycosylation-deficient NLGNs; GAPDH was used as a loading control.

E-H. Cells were permeabilized using Triton X-100, immunolabeled for both surface + intracellular HA-epitopes (i.e. total NLGNs), a golgi-complex marker GM130, and nuclear DAPI stain. Sample images (left) and average percentages (Mander's co-efficient, right) of HA-tagged NLGN1 (**E**), NLGN2 (**F**), NLGN3 (**G**), and NLGN4 (**H**), WT vs. glycan-null mutants associated with GM130. Orange vs. cyan arrowheads point at golgi apparatus co-localized with HA-NLGNs of transfected cells (green arrows) or from untransfected neighbors (white arrows).

Average values indicate means ± SEM, with individual data-points as color-matched filled circles. Numbers on bar-graphs denote total number of batches tested or field-of-views analyzed / independent biological replicates.

Statistical significances were measured using either two-tailed, unpaired, Student's t-test (for panels **A-D**), or two-sided, Mann-Whitney U-test (for panels **E-H**), with *** $P < 0.005$; * $P < 0.05$; ns = not significant, $P > 0.05$.

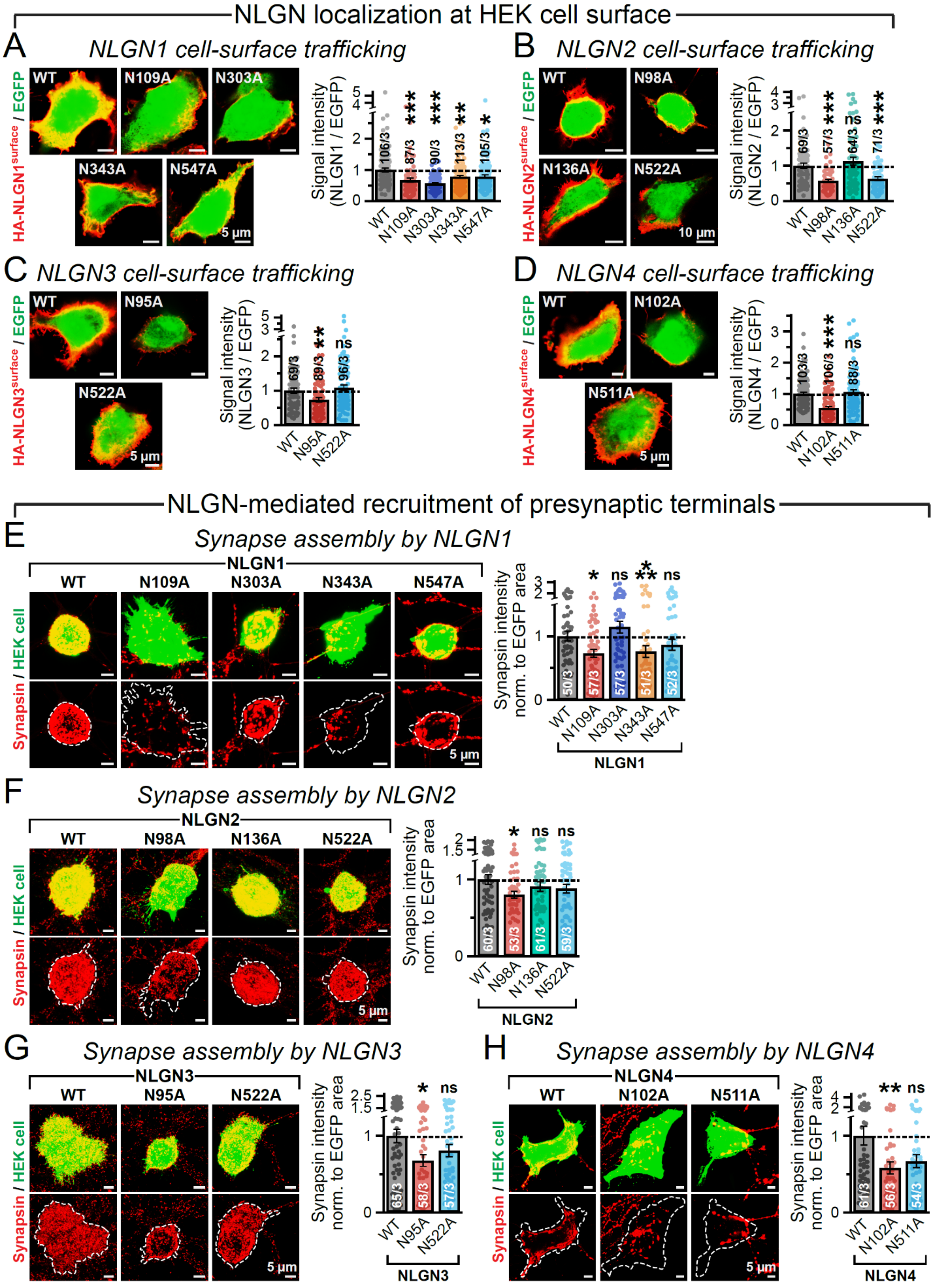

Supplementary Figure S5: **Glycan mutations impair NLGN-mediated assembly of presynaptic terminals.**

HEK cells expressed HA-tagged NLGN WT vs. single-site glycan mutants, followed by IRES-EGFP constructs. Cells were also co-cultured with primary neurons (see Fig. **4G**), and tested for their ability to recruit synapses.

A-D. Cells were immunostained for surface HA-epitopes under non-permeabilized condition. Example images (left, ≈ 0.5 μm optical thickness) and surface NLGN signals normalized to soluble EGFP levels (right) of HEK cells expressing HA-tagged NLGN1 (**A**), NLGN2 (**B**), NLGN3 (**C**), and NLGN4 (**D**), WT vs. single-site mutants.

E. Representative images (left) of EGFP-labeled HEK cells co-expressing NLGN1 variants (top panels), and surrounded by Synapsin-positive presynaptic specifications from adjacent primary neurons (coinciding within dotted boundaries, bottom panels); average Synapsin intensities (right) from different conditions, as indicated.

F-H. Same as **E**, except for WT vs. single-site glycan mutants of NLGN2 (**F**), NLGN3 (**G**), and NLGN4 (**H**).

All numerical data are means ± SEM, with total number of cells analyzed / number of independent trials (insets on bar-graphs) and individual data-points (color-matched filled circles). Statistical significances were calculated by two-sample, Mann-Whitney U-test, with *** $P < 0.005$; ** $P < 0.01$; * $P < 0.05$; ns = not significant, $P > 0.05$.
